# No evidence of attentional prioritization for threatening targets in visual search

**DOI:** 10.1038/s41598-024-56265-1

**Published:** 2024-03-07

**Authors:** Andras N. Zsido, Michael C. Hout, Marko Hernandez, Bryan White, Jakub Polák, Botond L. Kiss, Hayward J. Godwin

**Affiliations:** 1 Institute of Psychology, University of Pécs, 6 Ifjusag Street, Pécs, 7624 Baranya Hungary; 2 Szentágothai Research Centre, University of Pécs, Pécs, Hungary; 3 Department of Psychology, New Mexico State University, Las Cruces, USA; 4 Department of Economy and Management, Ambis University, Prague, Czech Republic; 5 Faculty of Science, Charles University, Prague, Czech Republic; 6 School of Psychology, University of Southampton, Southampton, UK

**Keywords:** Visual search, Snake, Threat detection, Visual feature, Affective feature, Negative valence, Psychology, Human behaviour

## Abstract

Throughout human evolutionary history, snakes have been associated with danger and threat. Research has shown that snakes are prioritized by our attentional system, despite many of us rarely encountering them in our daily lives. We conducted two high-powered, pre-registered experiments (total N = 224) manipulating target prevalence to understand this heightened prioritization of threatening targets. Target prevalence refers to the proportion of trials wherein a target is presented; reductions in prevalence consistently reduce the likelihood that targets will be found. We reasoned that snake targets in visual search should experience weaker effects of low target prevalence compared to non-threatening targets (rabbits) because they should be prioritized by searchers despite appearing rarely. In both experiments, we found evidence of classic prevalence effects but (contrasting prior work) we also found that search for threatening targets was slower and less accurate than for nonthreatening targets. This surprising result is possibly due to methodological issues common in prior studies, including comparatively smaller sample sizes, fewer trials, and a tendency to exclusively examine conditions of relatively high prevalence. Our findings call into question accounts of threat prioritization and suggest that prior attention findings may be constrained to a narrow range of circumstances.

## Introduction

A large body of prior studies^1–8^ have shown that emotion-evoking items (like threats) tend to capture and hold attention more readily than neutral ones. This is an adaptive feature of the visual system, and it is especially true regarding animal threats, such as snakes, which have posed a (real or perceived) survival threat throughout mammalian evolution^9–11^. For instance, the detection of spiders has been shown to be little affected by inattentional blindness even if presented in the periphery^11^, and snakes are efficiently detected even under challenging visual conditions^12–14^. This rapid detection of snakes could be explained by a combination of their emotion-evoking and visual characteristics. Snakes elicit high levels of arousal (compared to neutral stimuli) and therefore are more prone to automatically capture attention (and are harder to inhibit; Zsido et al.^15,16^). Further, the human visual system seems to prioritize the processing of curvilinear shapes (like the body of a snake) compared to rectilinear ones^17^, downward-pointing V-shapes (like the head of a snake) compared to upward-pointing ones^18^, and snakeskin-like textures compared to lizard skins and bird plumage^19^. It has been shown that the detection of both personally and biologically relevant stimuli, such as emotional stimuli and fear-related information in particular, are prioritized via the amygdala^20–22^. In fact, the *relevance superiority effects* hypothesis^23^ posits that perceived personal relevance (e.g. objects that are relevant for survival regardless of affective value) may be critical to the emotional and cognitive impacts of threatening information.

However, there exist two common shortcomings of past research that we sought to address in the present study. First, in contrast to the very large number of objects in our environment, past studies have used sparsely populated displays with only a small number (i.e. 4 to 9) of stimuli present^9,18,24,25^. While these studies are often described by researchers as being visual search tasks (or being comparable to visual search tasks), in reality, they utilize an “odd-one-out” paradigm in which a small number of objects are presented, and participants are asked to decide if one of the images belongs to a different category of objects (e.g. one snake among three flowers) or if all images belong to the same category (e.g. four flowers). Such displays are also typically highly organized, with the objects arranged in a circular or grid pattern. This sort of task, and the displays used therein, are therefore vastly different from visual search tasks that use a large number of objects randomly scattered over the screen, and that require participants to deliberately search for a pre-specified target, rather than to determine if one (unspecified) item is a mismatch to the others (see Hout and Goldinger^26^).

Another important concern is that although most of us only encounter snakes very rarely in our daily lives, past research has examined the attentional prioritization for threats typically by presenting them on a large proportion of trials, with prevalence rates falling between 50 and 100%^4,5,19,27^. As we describe below, the proportion of trials on which a target appears is known to have a significant influence upon search behavior and performance. We assume that the general tendency to present targets on a large proportion of trials is a result of researchers being keen to maximize their statistical power (i.e. by presenting targets as often as possible). However, the consequence of this approach is that it reduces ecological validity significantly by allowing for habituation to (or priming of) the stimuli across trials. We have summarized these two issues in Table 1, which presents a summary of many recent studies in this area, charting how they compare to our current two experiments.

**Table 1:** A list of previous studies investigating the advantaged attentional processing of threats (e.g. snakes, guns), reporting number of participants, number of trials, objects appearing on the display per trial, task type, and prevalence of a threat appearing.

Experiment	Participant count	Trial count	Objects per trial	Task type	Threat prevalence
Armstrong et al.^24^	65	24	4	Odd-one-out	100%
Fox et al.^23^					
Experiment 1	38	192	4	Odd-one-out	50%
Experiment 2	30	192	4	Odd-one-out	50%
Experiment 3	22	192	4	Odd-one-out	50%
Experiment 4	36	192	4	Odd-one-out	50%
Gao et al.^9^	64	144	9	Odd-one-out	100%
	32	288	9	Odd-one-out	100%
Larson et al.^18^	28	120	3	Odd-one-out	33%
LoBue^17^					
Experiment 1	48	24	9	Odd-one-out	100%
Experiment 1a	48	24	9	Odd-one-out	100%
Experiment 1b	48	24	9	Odd-one-out	100%
Experiment 2	96	48	9	Odd-one-out	100%
Experiment 3	96	48	9	Odd-one-out	100%
LoBue and Matthews^4^					
Experiment 1	27	128	4 or 9	Odd-one-out	100%
Experiment 2	24	128	4 or 9	Odd-one-out	100%
Experiment 3	21	128	4 or 9	Odd-one-out	100%
March et al.^7^	107	384	8	Odd-one-out	50%
Soares et al.^12^					
Experiment 1	57	288	4, 6, or 8	Odd-one-out	50%
Experiment 2	42	288	4, 6, or 8	Odd-one-out	50%
Experiment 3	57	288	4, 6, or 8	Odd-one-out	50%
Experiment 4	49	288	4, 6, or 8	Odd-one-out	50%
Öhman et al.^5^					
Experiment 1	25	144	4 or 9	Odd-one-out	50%
Experiment 2	30	256	4 or 9	Odd-one-out	50%
Experiment 3	34	256	4 or 9	Odd-one-out	50%
Van Strien and Isbell^19^	24	900	6	Odd-one-out	50%
Zsido et al.^8^	53	208	4 or 9	Odd-one-out	50%
Our study					
Experiment 1	115	300	32	Visual search	10% or 50%
Experiment 2	109	300	32	Visual search	10% to 50%

Why is it important that we consider the proportion of trials on which a target is presented? A large body of previous research has demonstrated that when target prevalence is low, participants rapidly respond ‘absent’ when searching, causing them to miss targets when they finally do appear^28–31^. Moreover, even when directly examined, searchers tend to miss target objects that are rare^28,29^. Current theory holds that low prevalence rates induce a criterion shift in decision-making, such that only the most strikingly visible and/or prototypical objects from a category are detected in these conditions^32^. Indeed, it has been shown that atypical members of target categories are more likely to be missed than typical members in a variety of circumstances^33,34^. In light of these findings, we reasoned that if threats (e.g. snakes) are conferred with an attentional prioritization during search, then they should experience weaker effects of target prevalence compared to non-threating targets (e.g. rabbits). Put another way, past research in this area tells us that threatening targets should be more easily detected than non-threatening ones, even when they only rarely appear^35^.

We tested this idea using two experiments that addressed the following question: does the attentional prioritization to threats provide them with some level of protection against the effects of low target prevalence? In both experiments, we manipulated overall target prevalence (i.e. a target appeared on either 10% or 50% of trials). In Experiment 1, targets could be threatening (snakes) or neutral (rabbits) items. Experiment 2 was a replication and extension of Experiment 1; besides threatening (snakes) and neutral (rabbits) targets, we also used negative but nonthreatening (cockroaches) and neutral but visually similar (to threats) targets (caterpillars). Our overarching goal was to test if threatening targets are found faster in a visual search paradigm that is more ecologically valid and complex than the “classical” ones used in prior literature. Our first hypothesis was that threatening targets would be detected more rapidly and would exhibit higher accuracy rates compared to neutral ones. Our second hypothesis was that task performance for finding threatening targets would be less impacted by the prevalence effect than non-threatening ones.

## Experiment 1

In Experiment 1, participants were engaged in a visual search task of looking for either threatening (*snakes*) or neutral (*rabbits*) targets among mixed real-world distractors (e.g., a balloon, a lamp, a dog). Targets appeared at either low prevalence (10% of trials) or high prevalence (50% of trials). In addition to traditional behavioral measures such as response time and accuracy (proportion of correct responses), we computed Balanced Integration Scores (Liesefeld and Janczyk^36^) which aim to control for potential speed-accuracy trade-offs that are common in visual search tasks. Balanced Integration Scores (BIS) integrate RTs and accuracy to provide a measure of relative performance^36,37^. We predicted that threatening targets would be detected more rapidly and would exhibit higher accuracy rates compared to neutral ones. In line with previous research^31^, we predicted that target detection rates and target-absent trial RTs would be lower for low prevalence targets than high prevalence ones. Further, we hypothesized that the difference in performance between the low and high prevalence conditions would be smaller for those who searched for threats compared to neutral targets.

### Method

#### Participants

We conducted an a priori power analysis using G*Power^38^. The estimated required total sample size (f = 0.40, power = 0.95, numerator df = 3) was 112. We attempted to recruit up to 120 participants, anticipating that not all of them would complete the entire task. A total of 119 students from the University of Pécs participated. All participants were compensated for their efforts with a reward of 5000 HUF (approximately 15 USD).

All participants reported normal or corrected-to-normal vision. Our research was approved by the Hungarian United Ethical Review Committee for Research in Psychology and was carried out in accordance with the Code of Ethics of the World Medical Association (Declaration of Helsinki). All participants provided written informed consent. The study was preregistered at OSF (https://osf.io/3nbtp).

#### Design

We used a 2 × 2 design with Prevalence (low, high) and Target Type (nonthreatening, threatening) as between-subject factors. Participants were assigned to conditions in counterbalanced order. There were 28 participants in the low prevalence nonthreatening and high prevalence threatening conditions, 29 participants in the high prevalence nonthreatening condition, and 30 participants in the low prevalence threatening condition.

#### Stimuli

Rabbit (nonthreatening target) images were sourced from Internet searches and snakes (threatening targets) were taken from a previously validated database^39^. Distractors were photographs of real-word objects from the Massive Memory database^40,41^. None of these stimuli had a background, unlike past studies in this area. The images were resized to approximately the same size (i.e., no larger than 100 × 100 pixels) maintaining original proportions. We used a large number of distractors (i.e., 240 categories with 15–16 exemplars per category) and targets (30 exemplars per category) that were randomly sampled across trials (and participants) to ensure that distractors and targets were comparable, and to reduce the possible nuisance effects of low- and mid-level visual features of the individual objects.

**Figure 1:**
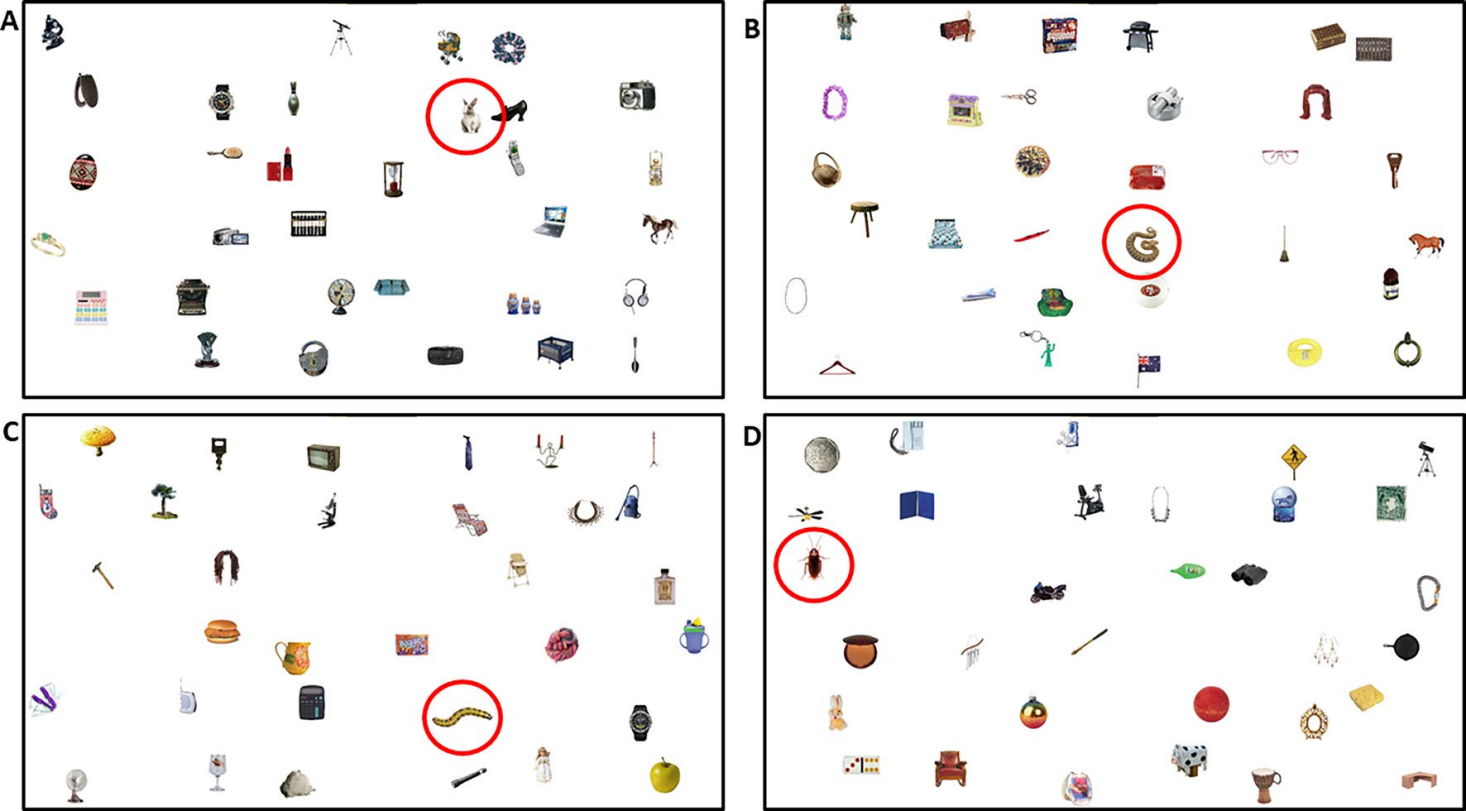
Sample trials showing the search array used in the two experiments. (**A**,**B**) Shows targets sampled from Experiment 1 (rabbit and snake conditions, respectively), (**C**,**D**) shows targets from Experiment 2 (caterpillar and cockroach conditions, respectively). Please note that while we highlighted targets with red circles for better visibility here, they were not used during the experiment.

A search array (see Fig. 1) algorithm created spatial configurations with a pseudorandom organization following previous research^26,29,42,43^. This algorithm breaks the display down into a virtual 6 × 6 grid (visual angle of width and height of grid cells were 7.99° × 4.95°). Eight objects were placed in each quadrant (i.e., one per cell); visual angle of width and height of objects were 2.53° × 2.53°. One of the 9 cells per quadrant was always left blank to make the displays appear more ‘random’ and less organized. The location of each object within their cells was randomly jittered. The target appeared in each quadrant of the display on an almost equal number of trials; 7 in two quadrants and 8 in the other two quadrants, (counterbalanced across participants in the same condition). For each trial, distractors were selected in a quasi-random sequence such that only one exemplar per category could appear; all categories were cycled through evenly across trials.

#### Apparatus and procedure

Participants were engaged in the study in small groups on up to 6 computers simultaneously (with identical hardware and software profiles) in a computer room. Participants were seated in separated workstation booths, at approx. 60 cm in front of 21.5-inch LCD monitors with a resolution of 1920 × 1080, 16:9 aspect ratio, a refresh rate of 60 Hz, and a color depth of 16.7 M. Stimuli were presented and randomized using E-Prime vs3 software (Psychology Software Tools, Pittsburgh, PA^44^). Experimental sessions were monitored by one research assistant. Participants started the task after being given detailed verbal and written instructions, as well as an opportunity to ask any questions of clarification.

The experiment began with 20 practice trials (at the same prevalence rate as the experimental trials) and participants received feedback on whether their answer was correct or not. These trials were not analyzed. This was followed by three blocks of 100 experimental trials where we only provided block-level feedback (i.e., percentage of correct answers) to participants. The target present and target absent trials were randomly distributed across the 100 trials within each block.

Each trial started with a black fixation cross on a white background, which was presented for 500 ms. Then the search array was presented, where participants were instructed to react as quickly and accurately as possible and press the spacebar when they decided whether the target was present or absent. After pressing the spacebar, a question appeared on an otherwise blank screen asking participants to report if they saw the target or not. They reacted by pressing either the A (‘I saw the target’) or the L (‘I did not see the target’) button on the keyboard. We used this response method to more accurately measure reaction times and accuracy and to avoid mistakes stemming from mixing up the key responses^42^. Participants were allowed to take a short break between the experimental blocks if they felt it was necessary. One session of data collection lasted approximately 30 to 45 min.

### Results

#### Analytic approach

We identified and removed outlier trials, defined as those under 250 ms (less than 1% of all trials) or those more than 2.5 standard deviations longer than the group mean (less than 3% of trials). Four participants were excluded from our analyses for having mean accuracies that were > 2.5 standard deviations below their group mean or mean RTs > 2.5 standard deviations above their group mean. The final sample size that was used in the analyses was 115. We computed BIS scores by (for each participant and condition) subtracting the standardized RT from the standardized proportion correct (PC) values (BIS = zPC–zRT). Lower BIS scores indicate less “efficiency” relative to other conditions/groups.

We slightly deviated from the preregistered analysis plan (https://osf.io/3nbtp) upon the request of the reviewers. Instead of performing a 2 × 2x2 ANOVA with Target absence, Prevalence, and Target type as factors, we performed 2 × 2 ANOVAs to test the effects of Prevalence (low, high) and Target Type (snake, rabbit) on performance (indicated by accuracy, RTs, and BIS). That is, target present and absent trials were analyzed separately. For the sake of transparency and consistency, we report the results of the original analysis plan in Supplementary material 1. Only correct trial RTs were analyzed. Statistical results are presented in tables instead of in text to make the description of the results easier to follow. See Supplementary material 2 for the detailed descriptive statistics including accuracy, RT, and BIS across all conditions.

#### Accuracy

**Figure 2:**
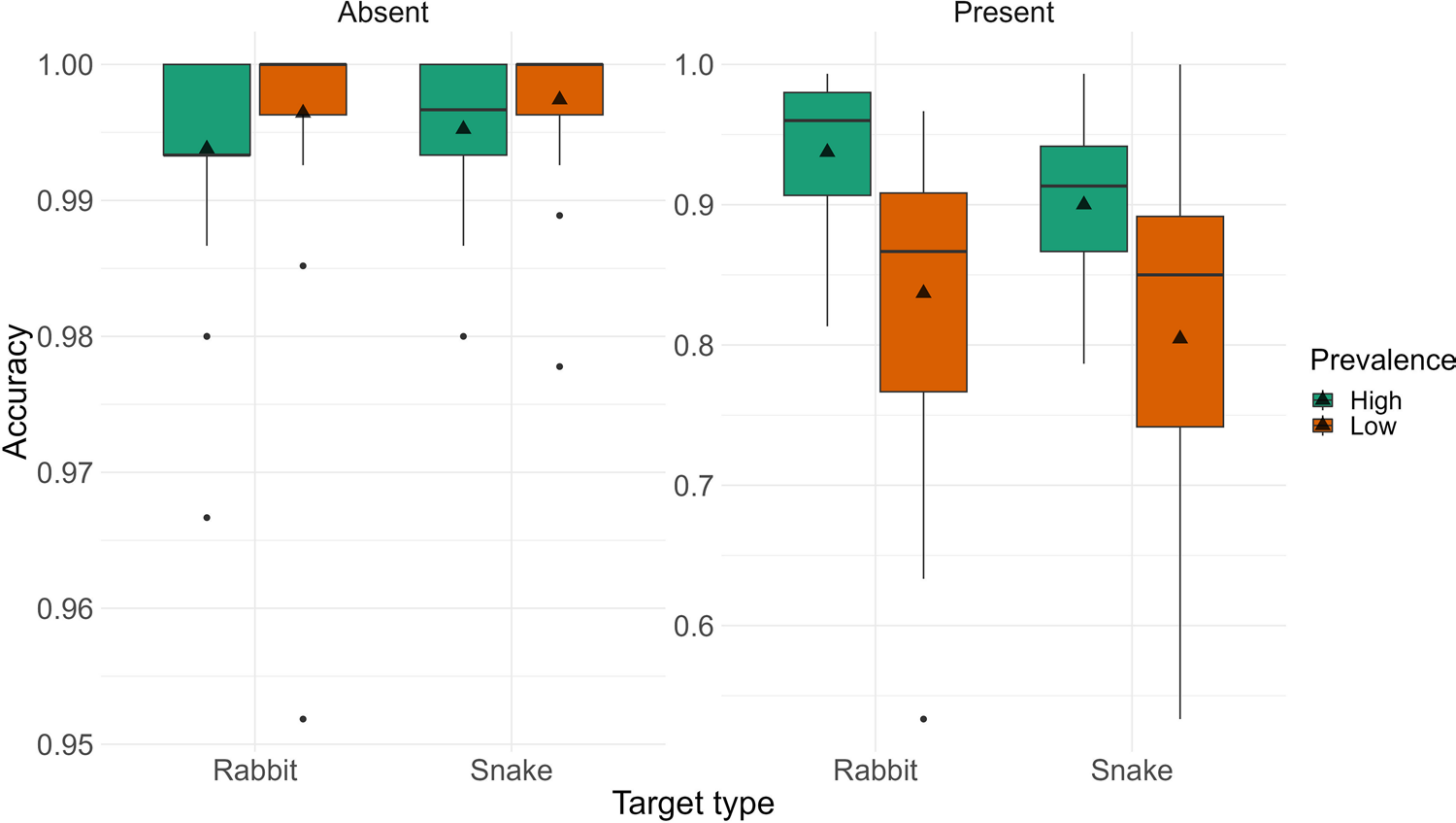
Accuracy in Experiment 1 for low and high prevalence items, and rabbit (neutral) and snake (threatening) targets, visualized as boxplots (separately for target-present and target-absent trials). In all boxplots presented in this paper, the line in the box represents the median, the triangle represents the mean, and the individual dots are outliers. Note that outlier in a box plot falls beyond 1.5 times the interquartile range.

We began by examining response accuracy to test our prediction that the prevalence effect would be weaker for snakes compared to rabbits. Figure 2 presents the descriptive statistics for these comparisons; see Table 2 for the statistical results. For target present trials, our ANOVA revealed a main effect of Prevalence; all other effects and interactions were not significant. We replicated the standard effect of target prevalence, as participants were less accurate in the low compared to the high prevalence condition. Although snake targets were found with slightly lower accuracy than rabbits (at the level of the means) in target-present trials, the pairwise comparison did not reach significance (*p* = 0.059). For target absent trials, all effects were nonsignificant. Contrary to our predictions, response accuracy for the detection of snake targets was not higher than for rabbits.

**Table 2:** Detailed statistical results for Experiment 1 (accuracy, reaction time, and BIS) with main effects and interactions.

Measure	Effect	Target present				Target absent			
		df	F	p	η^2^p	df	F	p	η^2^p
Accuracy	Prevalence	1, 111	28.5451	< 0.001	0.205	1, 111	3.1584	0.078	0.028
	Target type	1, 111	3.6293	0.059	0.032	1, 111	0.8037	0.372	0.007
	Prevalence * target type	1, 111	0.0186	0.892	0.000	1, 111	0.0297	0.863	0.000
RT	Prevalence	1, 111	9.877	0.002	0.082	1, 111	9.81	0.002	0.081
	Target type	1, 111	10.739	0.001	0.088	1, 111	5.03	0.027	0.043
	Prevalence * Target type	1, 111	0.438	0.510	0.004	1, 111	1.09	0.299	0.010
BIS	Prevalence	1, 111	47.46352	< 0.001	0.300	1, 111	10.47	0.002	0.086
	Target type	1, 111	10.51310	0.002	0.087	1, 111	4.85	0.030	0.042
	Prevalence * Target type	1, 111	0.00304	0.956	0.000	1, 111	1.27	0.262	0.011

#### RTs

**Figure 3:**
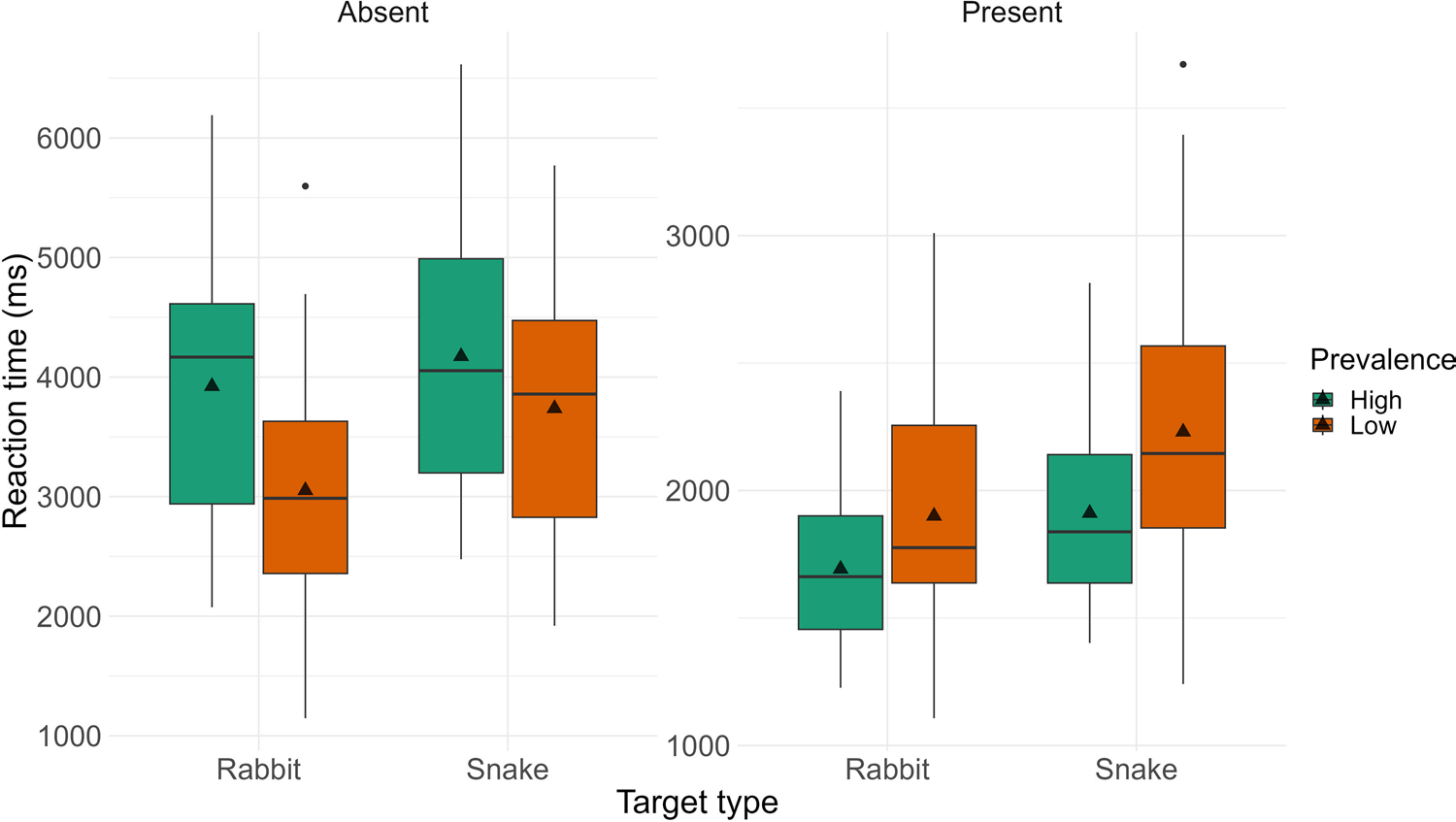
Reaction Times (in milliseconds) in Experiment 1 for low and high prevalence items, and rabbit (neutral) and snake (threatening) targets visualized as boxplots (separately for target-present and target-absent trials).

We next examined RTs, again to examine our prediction that the prevalence effect would be weaker for snakes compared to rabbits. Figure 3 presents the descriptive statistics for these comparisons; statistical results are presented in Table 2. For target present trials, the ANOVA revealed a significant main effect of Prevalence—replicating the standard prevalence effect—and Target Type. In contrast to our expectations, participants were *slower* to find snakes compared to rabbits. We found similar effects in target absent trials (i.e., the main effect of Prevalence and Target Type was significant). The interaction between the two factors were nonsignificant in both cases. Thus, while we found evidence for the standard effects of target prevalence, contrary to our predictions, performance in terms of RTs was worse for snakes compared to rabbits.

#### BIS

**Figure 4:**
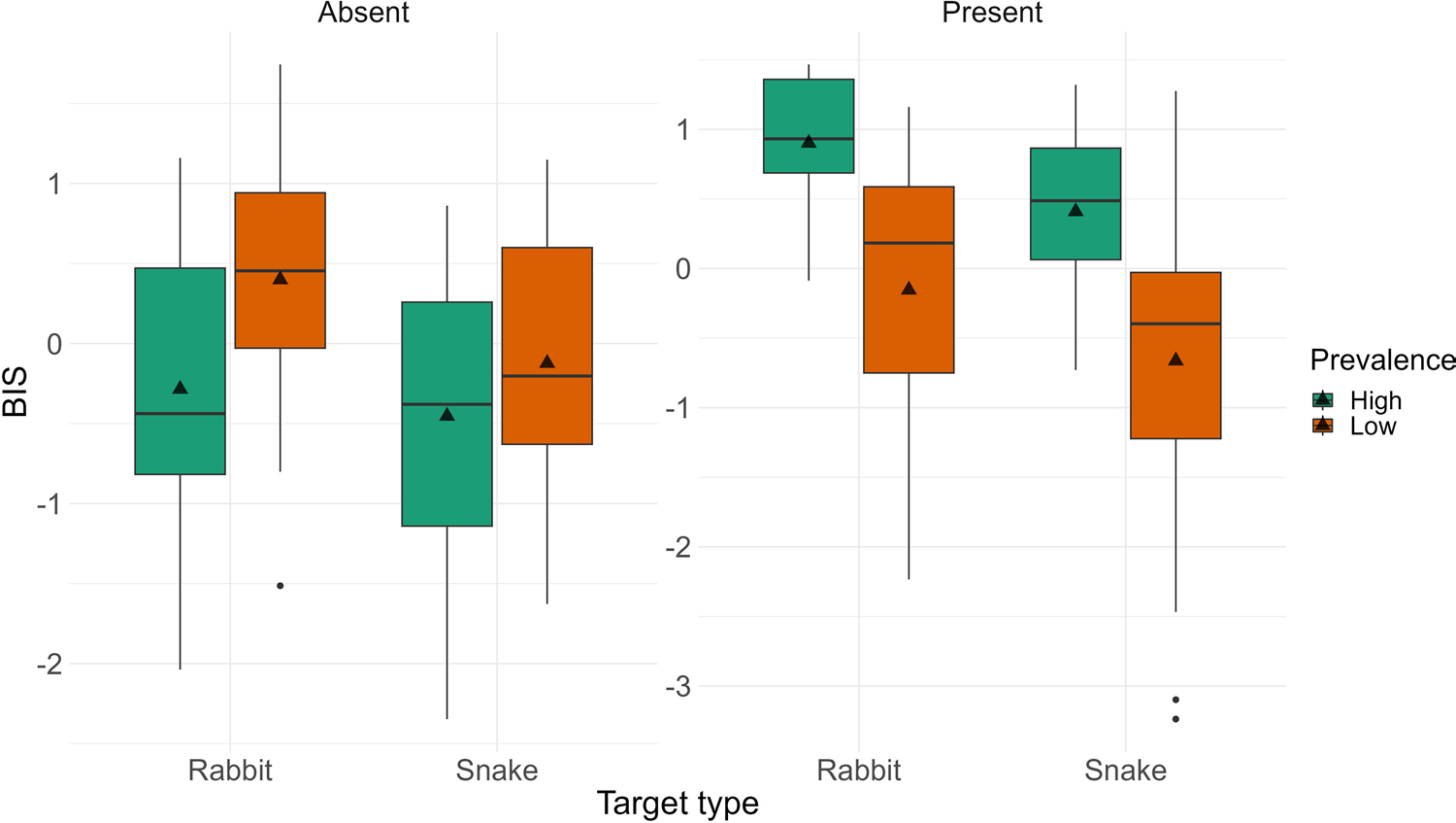
Balanced Integration Scores (BIS) in Experiment 1 for low and high prevalence items, and rabbit (neutral) and snake (threatening) targets visualized as boxplots (separately for target-present and target-absent trials).

Finally, we examined the efficiency of performance (using BIS scores) to test our prediction that prevalence effects would be less pronounced for snake compared to rabbit targets. Figure 4 presents the descriptive statistics for these comparisons; Table 2 shows all statistical results. For both target present and absent trials, we found that the main effect of target Prevalence was significant; finding targets in the low prevalence condition was less efficient compared to finding them in the high prevalence condition. The main effect of Target Type was also significant. Contrary to our prediction, finding snakes was less efficient compared to rabbits. The interactions were nonsignificant.

### Discussion

The main goal of Experiment 1 was to determine whether threatening targets showed weaker prevalence effects compared with non-threatening ones. Overall, we found evidence of standard prevalence effects^31^: that is, participants were more likely to miss low prevalence targets than high prevalence ones. Moreover, target-absent RTs were more rapid for low prevalence than high prevalence searchers. Surprisingly, however, we found no evidence of differences in response accuracy for snake versus rabbit targets, which contrasts with our predictions. In addition to this, participants were slower to detect snake targets compared to rabbits. Both results fail to provide evidence for any form of attentional prioritization of threatening targets, and the BIS analysis (combining speed and accuracy) further validates this conclusion.

Before we can dive into the discussion of the possible theoretical explanations behind these counterintuitive results, we need to rule out a more banal explanation concerning the stimuli that we used. Although we have used a large number of distractors and targets that were randomly selected across participants to make distractors and targets comparable, and to reduce the possible nuisance effects of low- and mid-level visual features, it is possible that the shape of the snakes made their detection more difficult compared to those of the rabbits, and that that is the reason for our unexpected results. This explanation is unlikely, given that prior literature suggests that snakes should be found more easily/quickly, not less; however, we next sought to rule out stimulus idiosyncrasies or flukish results as an explanation for what we observed. For the dual purposes of replication and to rule out stimulus idiosyncrasies, (and also to test whether the visual or affective features of the snake caused this pattern of results), we therefore conducted a second experiment.

## Experiment 2

In Experiment 2, participants performed the same visual search task as in Experiment 1. Here, in addition to threatening (*snake*) and neutral (*rabbit*) targets, we also included groups who searched for negatively valenced but nonthreatening (*cockroach*) and neutral but visually similar to threat (*caterpillar*) targets. Representatives of categories were determined based on a previous study^45^. This was necessary to address the concern that remained after Experiment 1 that stimulus features were responsible for our results. Our modified design thus allowed us to explore threat relevance (snakes vs other targets), affective value (snakes and cockroaches vs neutral targets), and visual features (snakes and caterpillars vs other targets). We predicted that if threatening targets were prioritized by attentional systems due to one of these stimulus features (threat relevance, affective value, or visual form), then the effects of target prevalence should be reduced for the targets with those features compared with the ones that lack the feature. For instance, if snakes are prioritized due to their threat relevance, we should only find prioritization (e.g., better accuracy, faster search) for snakes but not for other negatively valenced or visually similar targets. Alternatively, if snakes are prioritized due to their visual form, we should find prioritization for both snakes and caterpillars, but not for other negatively valenced targets.

### Method

#### Participants

A total of 113 students from New Mexico State University participated for partial course credit. The estimated required total sample size for ANOVA with fixed effects, main effects, and interactions using the following parameters f = 0.40, power = 0.95, numerator df = 3, nr. of groups = 8 is 112; that is 14 participants per group.

All participants reported normal or corrected-to-normal vision and normal color vision. Our research was approved by the Institutional Review Board at New Mexico State University and was carried out in accordance with the Code of Ethics of the World Medical Association (Declaration of Helsinki). All participants provided written informed consent. The study was preregistered at OSF (https://osf.io/c8ae4).

#### Design and stimuli

We used a 2 × 4 design with Prevalence (low, high) and Target type (rabbit, snake, cockroach, caterpillar) as between-subject factors. There were 13 participants in the low prevalence rabbit, and low and high prevalence cockroach conditions, 14 participants in the low prevalence snake and high prevalence rabbit conditions, 11 in the high prevalence caterpillar condition, 16 in the high prevalence snake condition, and 15 participants in the low prevalence caterpillar condition. Negative nonthreatening (cockroaches) and visually similar to threat but neutral targets (caterpillars) were sourced from Internet searches. All other experimental details and the procedure of data acquisition were identical to Experiment 1.

#### Ethical approval

Ethics approval was obtained from dedicated Ethical Review Committees for Research in Psychology.

#### Informed consent

Informed consent was obtained from all individual participants included in the study.

### Results

#### Analytic approach

We first identified and removed outlier trials, defined as those less than 250 ms or greater than ± 2.5 standard deviations above the group mean (resulting in removal of less than 1% and 3% of all collected data for RTs that were too fast and too slow, respectively). Two participants were excluded from analysis for having mean RTs that were more than 2.5 standard deviations above their group mean, and two were dropped for self-reporting color blindness. This resulted in a final sample size of 109.

We performed 2 (Prevalence: low, high) × 4 (Target type: rabbit, snake, cockroach, caterpillar) ANOVAs to examine performance as indicated by accuracy, RTs, and BIS. Target present and absent trials were analyzed separately as in Experiment 1. Again, this is a slightly deviation from the preregistered analysis plan (https://osf.io/c8ae4) upon the request of the reviewers. For the sake of transparency and consistency, the results of the original analysis plan can be found in Supplementary material 1. Significant main effects were further analyzed by follow-up t-tests with Tukey correction. Statistical results are presented in tables instead of in text to make the description of the results easier to follow. Only correct trial RTs were analyzed. See Supplementary material 2 for the detailed descriptive statistics across all conditions.

#### Accuracy

**Figure 5:**
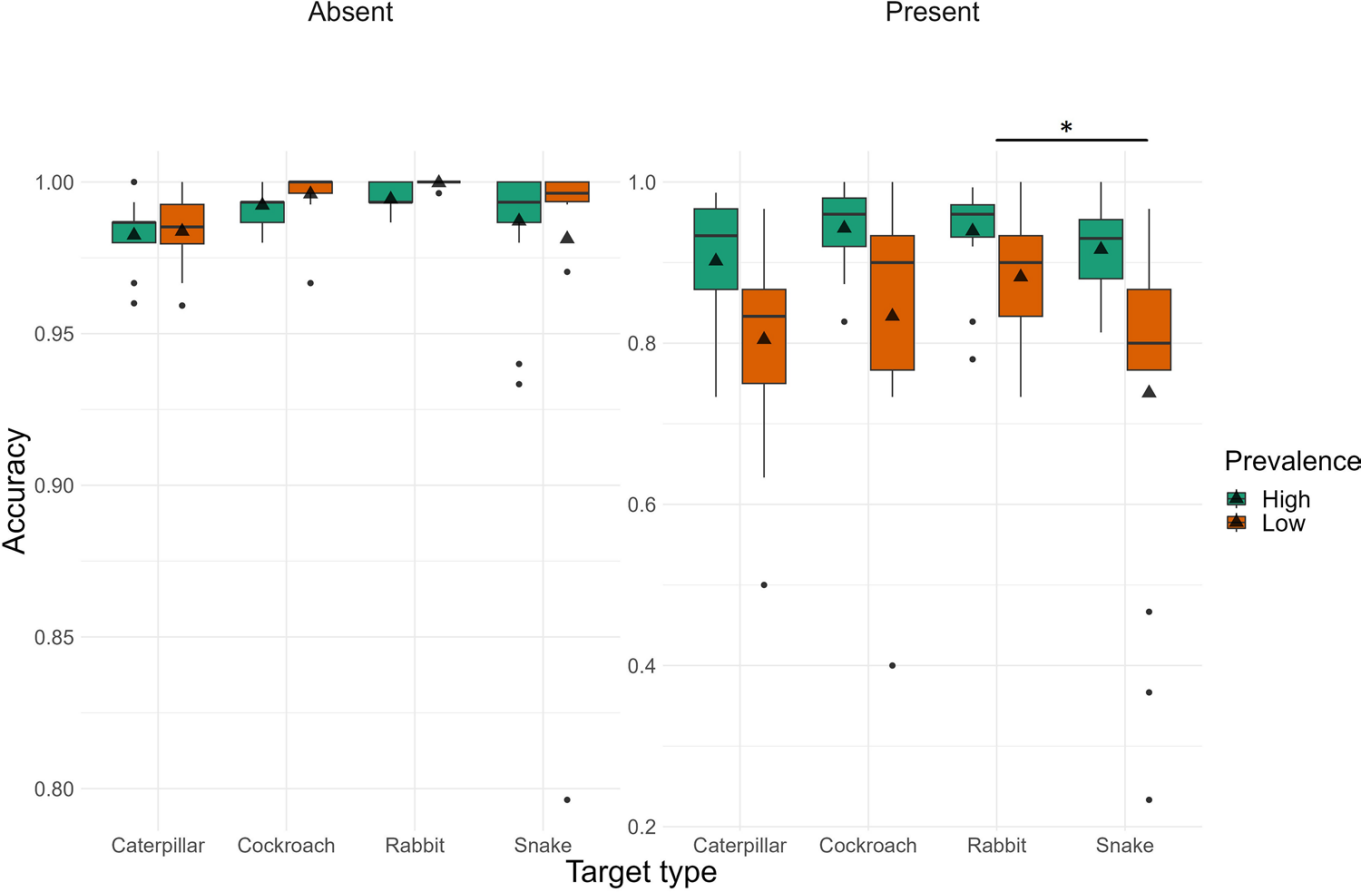
Accuracy in Experiment 2 for low and high prevalence items, and the four types of targets (separately for target-present and target-absent trials) visualized as boxplots.

We began by examining response accuracy to test our prediction that the prevalence effect would be weaker for threatening compared to nonthreatening targets. Figure 5 presents the descriptive statistics for these comparisons; see Table 3 for the statistical results. Regarding target present trials, our ANOVA revealed a main effect of Prevalence and of Target type. Replicating the results of Experiment 1, in target-absent trials, accuracy was similar across conditions, while in target-present trials, participants were less accurate in the low compared to the high prevalence condition. Further, participants identified snakes with significantly lower accuracy compared to rabbits, while cockroaches and caterpillars did not significantly differ from either of these categories. While the main effect of Target type was significant in target absent trials, the follow-up t-tests were nonsignificant. All other effects were nonsignificant. Contrary to our predictions, response accuracy for threatening targets was not higher than for nonthreatening ones.

**Table 3:** Detailed statistical results for Experiment 2 (accuracy, reaction time, and BIS) with main effects, interactions, and follow-up t-tests.

Measure	Effect	Target present				Target absent			
		df	F/t	p	η^2^p/ Cohen d	df	F/t	p	η^2^p/ Cohen d
Accuracy	Prevalence	1, 101	23.25	< 0.001	0.187	1, 101	0.0711	0.790	0.001
	Target type	3, 101	2.70	0.050	0.074	3, 101	2.7153	0.049	0.075
	Rabbit—Snake	101	2.640	0.047	0.701	101	2.186	0.134	0.5808
	Rabbit—Caterpillar	101	1.746	0.306	0.483	101	2.278	0.110	0.6299
	Rabbit—Cockroach	101	0.692	0.900	0.190	101	0.467	0.966	0.1284
	Snake—Caterpillar	101	− 0.809	0.850	− 0.218	101	0.182	0.998	0.0491
	Snake—Cockroach	101	− 1.906	0.232	− 0.511	101	− 1.687	0.336	− 0.4524
	Caterpillar—Cockroach	101	− 1.049	0.721	− 0.293	101	− 1.797	0.281	− 0.5015
	Prevalence * Target type	3, 101	1.28	0.284	0.037	3, 101	0.3623	0.780	0.011
RT	Prevalence	1, 101	5.47	0.021	0.051	1, 101	16.03	< 0.001	0.137
	Target type	3, 101	3.71	0.014	0.099	3, 101	2.95	0.036	0.081
	Rabbit—Snake	101	− 1.286	0.574	− 0.3415	101	0.460	0.967	0.122
	Rabbit—Caterpillar	101	0.361	0.984	0.0997	101	1.264	0.588	0.350
	Rabbit—Cockroach	101	1.990	0.199	0.5469	101	2.753	0.035	0.757
	Snake—Caterpillar	101	1.635	0.364	0.4413	101	0.842	0.834	0.227
	Snake—Cockroach	101	3.312	0.007	0.8884	101	2.365	0.091	0.634
	Caterpillar—Cockroach	101	1.602	0.382	0.4471	101	1.459	0.466	0.407
	Prevalence * Target type	3, 101	1.81	0.149	0.051	3, 101	1.35	0.262	0.039
BIS	Prevalence	1, 101	27.597	< 0.001	0.215	1, 101	15.64	< 0.001	0.134
	Target type	3, 101	4.133	0.008	0.109	3, 101	3.07	0.031	0.084
	Rabbit—Snake	101	2.972	0.019	0.7896	101	0.0488	1.000	0.0130
	Rabbit—Caterpillar	101	1.500	0.441	0.4148	101	− 0.751	0.876	− 0.2077
	Rabbit—Cockroach	101	− 0.036	1.000	− 0.0098	101	− 2.588	0.053	− 0.7114
	Snake—Caterpillar	101	− 1.388	0.510	− 0.3747	101	− 0.817	0.846	− 0.2206
	Snake—Cockroach	101	− 2.980	0.019	− 0.7994	101	− 2.701	0.040	− 0.7244
	Caterpillar—Cockroach	101	− 1.522	0.428	− 0.4247	101	− 1.805	0.277	− 0.5038
	Prevalence * Target type	3, 101	0.975	0.408	0.028	3, 101	1.18	0.323	0.034

#### RTs

**Figure 6:**
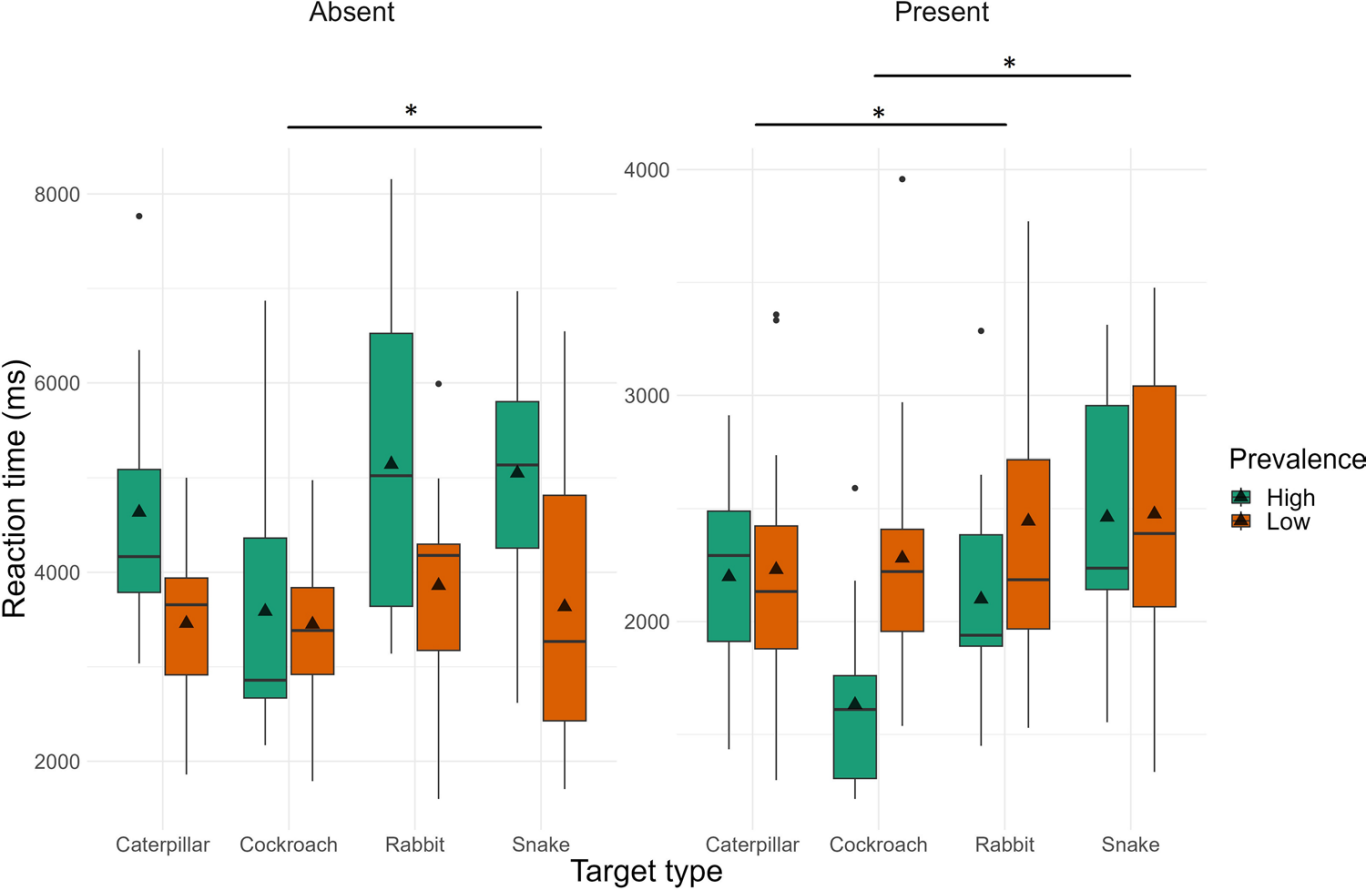
Reaction Times (in milliseconds) in Experiment 2 for low and high prevalence items and the four types of target (separately for target-present and target-absent trials) visualized as boxplots.

We next examined RTs, again to check for our predictions regarding the threat targets. Figure 6 presents the descriptive statistics for these comparisons; statistical results are presented in Table 3. Replicating the results of Experiment 1, for both target present and absent trials, the ANOVA revealed a significant main effect of Prevalence. RTs for targets in the low prevalence condition were higher compared to the high prevalence condition. The main effect of Target Type was also significant for both target present and absent trials; in contrast to our expectations, RTs did not differ for finding snakes and caterpillars and rabbits. Participants resolved search more quickly for cockroaches than snakes (in target present trials) and rabbits (in target absent trials). All other effects were nonsignificant. While we found evidence for the standard effects of target prevalence, again contrary to our prediction, performance was worse for threatening compared to nonthreatening targets.

#### BIS

**Figure 7:**
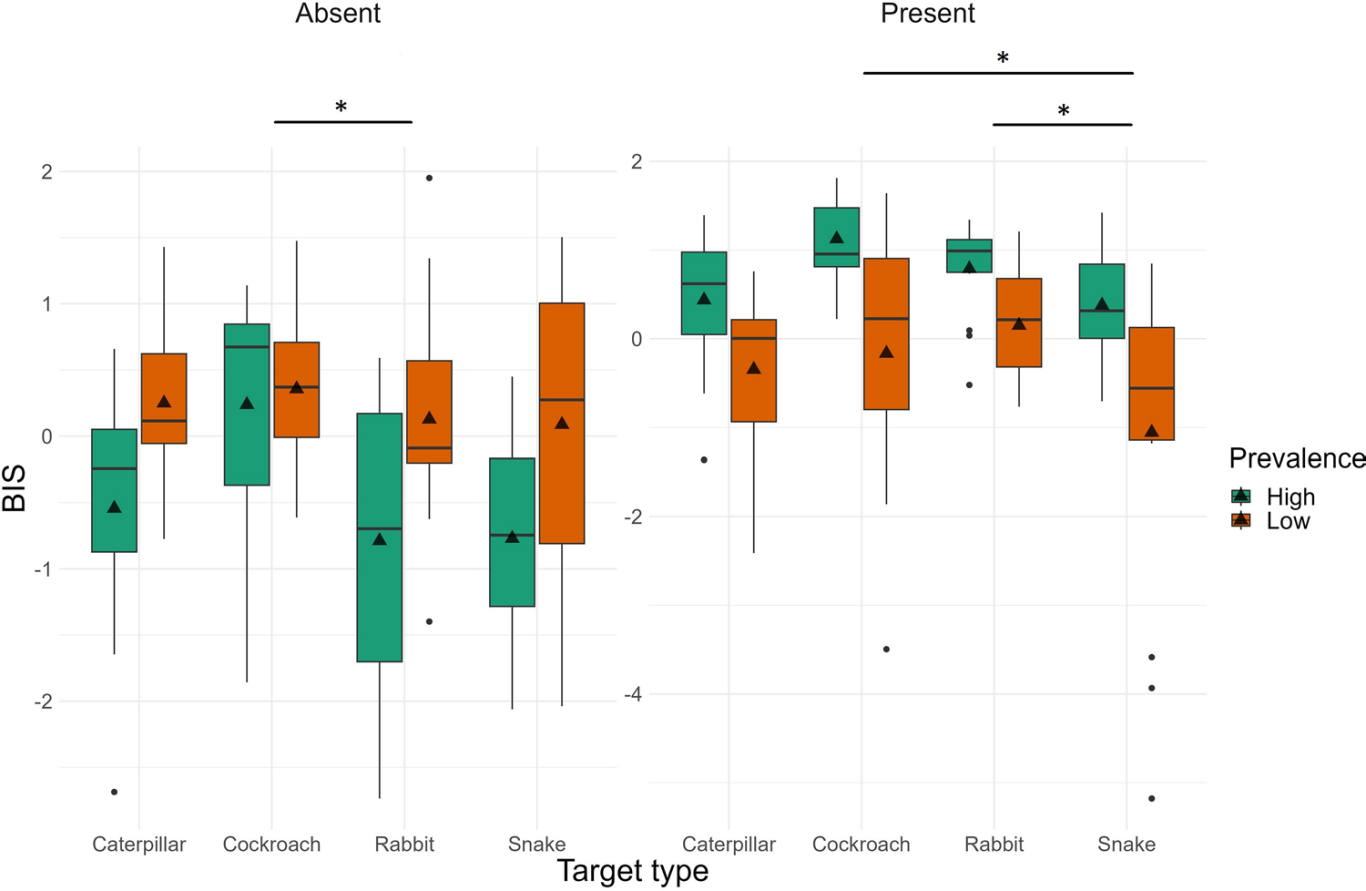
Balanced Integration Scores in Experiment 2 for low and high prevalence items and the four types of targets 9separately for target-present and target-absent trials) visualized as boxplots.

Finally, we examined efficiency of performance to test our prediction that this effect would be less pronounced for snakes compared to other nonthreatening targets (rabbits, cockroaches, and caterpillars). Again, replicating the results of Experiment 1, we found that the main effect of target Prevalence was significant regardless of target presence; performance in the low prevalence condition was less efficient compared to the high prevalence condition. The main effect of Target Type was also significant regardless of target presence. Contrary to our prediction, participants were less efficient finding snakes compared to rabbits and cockroaches in target present trials (see Fig. 7). In target absent trials participants were less efficient looking for snakes compared to cockroaches. All other effects were nonsignificant.

In sum, we found evidence for standard effects of target prevalence (in terms of accuracy and BIS, but not for RTs). Further, we replicated the results of Experiment 1; that is, participants’ performance was worse for threatening compared to nonthreatening targets. These results seem to support the notion that the effect seen in Experiment 1 is caused by both the threat relevance and visual characteristics of the targets. Further, negative valence alone (without threat relevance) does not seem to have an effect.

### Discussion

In Experiment 2, we replicated the results of Experiment 1; that is, search performance was worse in the low compared to the high prevalence condition, and finding threatening targets was harder compared to nonthreatening ones. This was true regardless of the fact that, given geographical differences, the participants recruited from southern New Mexico likely had a higher probability of encountering a snake in real life compared to the Hungarian sample in Experiment 1 (although to our knowledge, to date, there is no published data documenting such a difference). Thus, the effects we found in Experiment 1 are likely not due to the specific shape or a possible difference in the visibility of the targets. In contrast to Experiment 1, here we also found a difference in Target type; that is, while negative affective value did increase performance compared to neutral targets (as evidenced by the difference between cockroaches vs rabbits and caterpillars), threatening affective value decreased performance (compared to all other categories). This is, again, contradictory to what would be predicted by previous studies which suggested that threats are prioritized over neutral and other affective targets in visual processing^7,4,14^.

## General discussion

The goal of our two experiments was to investigate whether the attentional benefit to threats (as described in much prior research) conveys them with protection against prevalence effects. In Experiment 1, overall prevalence was manipulated whilst threatening and nonthreatening objects were visually dissimilar. In Experiment 2, we conceptually replicated our first experiment, adding to it target types that were neutral but emotionally salient (cockroaches), and neutral targets that were visually similar to the threat group (caterpillars).

In both of our experiments, we replicated past findings that lower prevalence targets were more likely to be missed than their higher prevalence counterparts^28,29,31^. We also found, again in line with past research, that target-absent RTs were reduced for low prevalence search (Experiment 1), and that lower-prevalence targets were found more slowly than higher prevalence ones (Experiment 2). These effects were not impacted by visual or affective properties of the targets, which is in line with the results of past studies on prevalence effects showing that target prevalence is a seemingly universal effect in the sense that it is not strongly affected by what the observer is looking for^35^﻿.

Surprisingly, and in contrast to both our predictions and a substantial body of existing research^46^, we found no evidence that threat targets benefited from any level of attentional prioritization during visual search. In Experiment 1, threatening targets were detected by participants more slowly than neutral targets. In Experiment 2, response accuracy was lower for the threatening targets than the non-threatening ones (including those with negative valence and those visually similar to threats). In both experiments, BIS scores indicated worse performance for the threatening targets compared to the neutral and nonthreatening ones. We did not find an interaction between the type of the target and target prevalence, indicating that the performance differences observed between various targets are similar across low and high prevalence rates. Again, this may indicate that attentional prioritization and prevalence effects seem to be independent of each other, though the present study was not designed to provide evidence for such a null effect.

In a previous study^15^, we have shown that people rely on visual information (such as shape) when that is enough to complete a task, and they only use affective information when the visual information is not enough to make a distinction. In Experiment 2, the nonthreatening and threatening targets were high in visual similarity, and the difference between them was expressed in terms of their affective value. Our new results showed that performance was decreased for threat targets compared to neutral and negative targets, while nonthreatening targets that were visually highly similar to threats did not differ from other conditions. Thus, it seems that somehow the threat relevance combined with the visual characteristics of the target slowed down responses. This is an unexpected result, as previous studies have generally found speeded response times for threatening compared to nonthreatening targets^1,3–6^. Our results might therefore be a first step towards challenging the current theories on threat perception.

When failing to find evidence of a classic and well-known finding, conflicting results that emerge pose a difficult question: Which set of results should be regarded as being the most accurate or reliable? Certainly, it is the case that the past studies (many of which have been listed in Table 1) generally agree with one another, so it is clear that the basic finding of attentional prioritization likely holds under the conditions that were being used by those researchers. Although it should also be noted that such results have previously been questioned, and mostly based on methodological grounds. For instance, a previous review^47^, empirical^48^ research^49^, and a meta-analytic study^50^ warn that past empirical support in favor of the prioritized detection and processing of threats is not convincing. And that brings us to the key point here: clearly, the conditions used by the researchers when studying attentional prioritization to threats is at least partially responsible for driving the divergent pattern of results between past research and ongoing studies like our own.

As noted above, and as summarized in Table 1, there are a number of key and important differences between our experiments and the past research in this area. First, we recruited many more participants than previous studies would typically recruit for both experiments (i.e., over a 100 for each, although the comparison is nuanced by the variety in number of groups in this and past studies). We also used a larger number of trials per participant compared with most previous studies (in both of our experiments). Because of these factors, we believe that the divergent pattern of results we have observed cannot be explained away as a failure to replicate on the basis of statistical power. If anything, our work here has a higher level of statistical power than previous research. Setting aside sample size comparisons, there are three key differences that can be drawn out which distinguish our experiments from prior research in this area. These key differences can all be drawn together as reflecting an increased level of ecological validity for our experiments compared with prior research.

First, we used more complex and less organized displays than prior research to achieve a higher level of ecological validity. As highlighted in Table 1, most prior research in this area presented participants with only a small number of objects per trial. It is therefore possible that the salience of threatening targets only allows them to “pop out” when there are a very small number of competitors/distractors in the array, and when the overall demands of the task are quite low due to low clutter and increased spatial organization (relative to our displays).

Second, we used a more standard visual search task rather than an odd-one-out task. Though related and similar in many ways, odd-one-out tasks have fundamental differences to visual search that could render threats more readily detectable by searchers. In the odd-one-out paradigm, participants often only need to decide if one of the images depicts a different category of objects (e.g., one snake among three flowers) or if all images are from the same category (e.g., four flowers). Visual search is a more intentional and directed task, requiring increased attentional focus and guidance because the observer is specifically looking for one particular object, rather than assessing global differences in the makeup of the items in a display.

Finally, we used low levels of target prevalence here, whereas previous research has used much higher levels of target prevalence. Our low prevalence searches were more similar to real-world threat detection wherein targets are very unlikely to appear often. It may in fact be the case that by using high prevalence in prior work, threats were unintentionally primed, making them easier to spot; this circumstance would have been less possible in our experiments with more reasonable prevalence rates.

It should be noted that while all target categories used here were animate, the majority of distractors were inanimate. The *animacy effect* proposes that animate objects are prioritized over inanimate ones in visual processing^51–53^. Given that all targets were animate, it is unlikely that animacy effects could pose a bias in the present study that would obscure prevalence effects or threat effects across target types. However, based on the prior documented animacy effects, it is likely that all targets were subject to attentional prioritization to some degree. This might have resulted in an overall better task performance (compared to other prevalence studies) and, consequently, may have attenuated the differences between target categories (i.e., observed effect sizes being smaller than the true differences would dictate).

In sum, our results demonstrate that under some circumstances, the detection of threatening objects may be harder than for nonthreatening objects (or at the very least, that threats are not universally prioritized in visual attention). The effect seems robust: we found the same general pattern of results in two high-powered studies, using independent samples collected in different countries. Future research is needed to explore the exact mechanisms underlying this unexpected pattern of results. For instance, eye-tracking and EEG studies could probe deeper into overt vs covert attention, providing us with finer-grained details about the sequence of events that unfold between the time of initial attentional capture, and behavioral response. Here, we were mostly concerned with visual search; subsequently the attentional prioritization effect of threats should be revisited in relation to other important attentional phenomena such as inattentional blindness^54,55^, or the enhanced incidental memory of objects encountered during multiple target search^56,57^. Nevertheless, our results are important insofar as they have practical relevance to the field of prevalence research, while calling into question seemingly well-established attentional phenomena that now clearly need to be reexamined.

### Supplementary Information

Supplementary Information 1.

Supplementary Information 2.

## Data Availability

The data that support the findings of this study are available from the OSF page of the study (https://osf.io/jbamv/).
