# Supplementary material for: No evidence of attentional prioritization for threatening targets in visual search
Source: Sci Rep. 2024 Mar 7;14:5651. doi: 10.1038/s41598-024-56265-1 (PMC10920919; doi:10.1038/s41598-024-56265-1)
Supplement: Supplementary file 1 — Supplementary Information 1. [file 41598_2024_56265_MOESM1_ESM.docx]

**Supplementary material 1**

Here we provide the results of a full analysis as presented in the preregistered analysis plan (<https://osf.io/3nbtp>) including target absent trials for both experiments. We used a 2x2x2 design with Prevalence (low, high) and Target Type (nonthreatening, threatening) as between-subject factors, and Target Presence (present, absent) as a within-subjects factor.

**Experiment 1**

*Accuracy*

We began by examining response accuracy both to replicate standard effects of target prevalence and to test our prediction that the prevalence effect would be weaker for snakes compared to rabbits. Figure 2 presents the descriptive statistics for these comparisons; see Table 2 for the statistical results. Our ANOVA revealed a main effect of Target Presence, of Prevalence, an interaction between Target Presence and Prevalence, and an interaction between Target Presence and Target Type. In target-absent trials, accuracy was similar across conditions, while in target-present trials, participants were less accurate in the low compared to the high prevalence condition. Accuracy was worse in target-present compared to target-absent trials for both prevalence rates and for both types of targets. Although snake targets were found with slightly lower accuracy than rabbits in target-present trials, the pairwise comparison did not reach significance (p=.059). Accuracy was equal for both type of targets in target-absent trials. All other effects were nonsignificant. Contrary to our predictions, response accuracy for snakes was not higher than for rabbits.


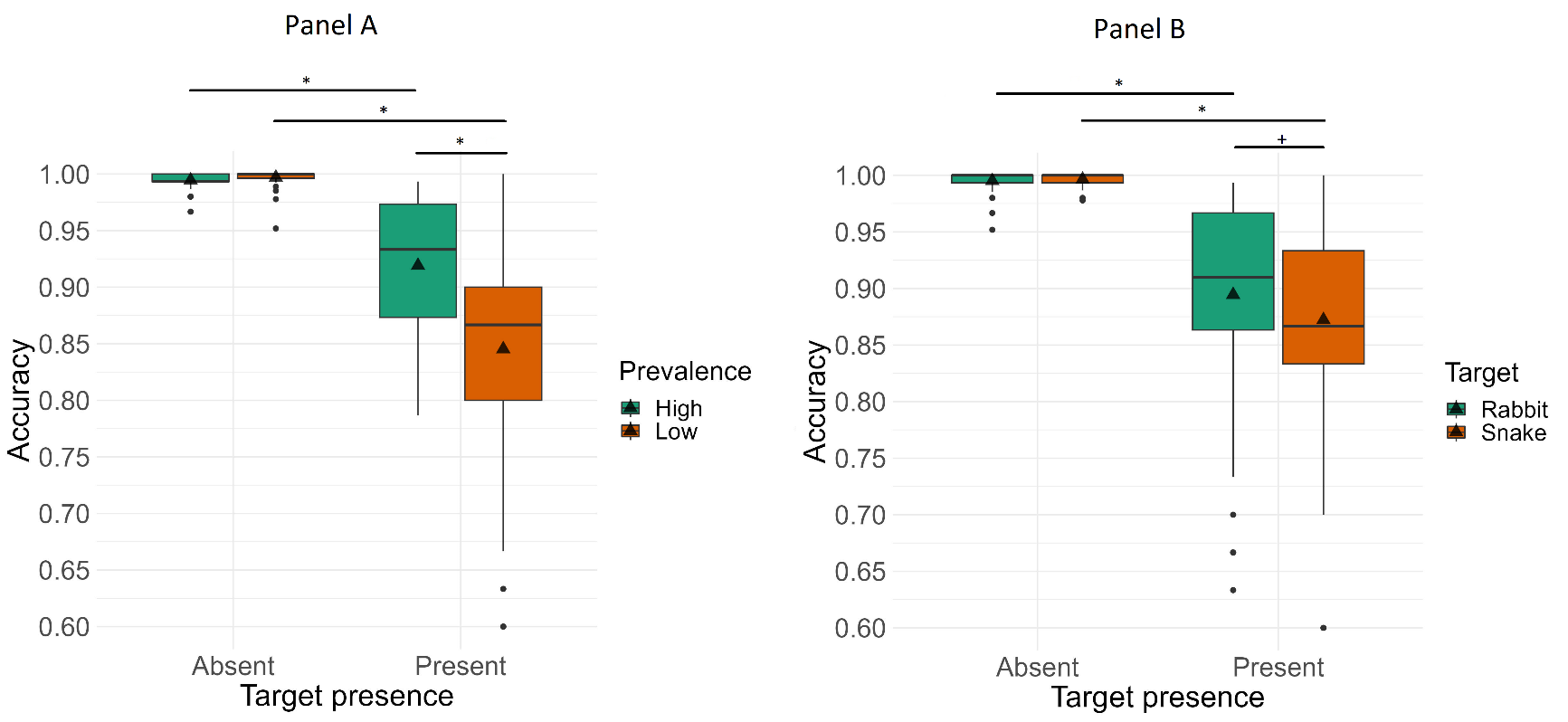


**Figure 2**. Accuracy in Experiment 1 for low and high prevalence items (panel A) and rabbit (neutral) and snake (threatening) targets (panel B) visualized as boxplots separately for target-present and target-absent trials.

**Table 2**. Detailed statistical results for Experiment 1 (accuracy, reaction time, and BIS) with main effects, interactions, and follow-up simple effects.

| **Measure** | **Effect** | **df** | **F** | **p** | **η²p** |
| --- | --- | --- | --- | --- | --- |
| **Accuracy** | Target presence | 1, 111 | 199.3063 | < .001 | 0.642 |
|  | Target presence ✻ Prevalence | 1, 111 | 31.6705 | < .001 | 0.222 |
|  | Absent Low - Absent High | 111 | 1.78 | 0.078 |  |
|  | Absent Low - Present Low | 111 | 14.02 | < .001 |  |
|  | Absent High - Present High | 111 | 5.98 | < .001 |  |
|  | Present Low - Present High | 111 | -5.34 | < .001 |  |
|  | Target presence ✻ Target type | 1, 111 | 4.1070 | 0.045 | 0.036 |
|  | Absent Rabbit - Absent Snake | 111 | -0.897 | 0.372 |  |
|  | Absent Rabbit - Present Rabbit | 111 | 8.515 | < .001 |  |
|  | Absent Snake - Present Snake | 111 | 11.463 | < .001 |  |
|  | Present Rabbit - Present Snake | 111 | 1.905 | 0.059 |  |
|  | Target presence ✻ Prevalence ✻ Target type | 1, 111 | 0.0235 | 0.878 | 0.000 |
|  | Prevalence | 1, 111 | 25.5097 | < .001 | 0.187 |
|  | Target type | 1, 111 | 3.1762 | 0.077 | 0.028 |
|  | Prevalence ✻ Target type | 1, 111 | 0.0144 | 0.905 | 0.000 |
|  |  |  |  |  |  |
| **RT** | Target presence | 1, 111 | 489.02 | < .001 | 0.815 |
|  | Target presence ✻ Prevalence | 1, 111 | 32.12 | < .001 | 0.224 |
|  | Absent Low - Absent High | 111 | -3.13 | 0.002 |  |
|  | Absent Low - Present Low | 111 | 11.68 | < .001 |  |
|  | Absent High - Present High | 111 | 19.56 | < .001 |  |
|  | Present Low - Present High | 111 | 3.14 | 0.002 |  |
|  | Target presence ✻ Target type | 1, 111 | 1.43 | 0.234 | 0.013 |
|  | Target presence ✻ Prevalence ✻ Target type | 1, 111 | 1.00 | 0.318 | 0.009 |
|  | Prevalence | 1, 111 | 2.030 | 0.157 | 0.018 |
|  | Target type | 1, 111 | 7.361 | 0.008 | 0.062 |
|  | Prevalence ✻ Target type | 1, 111 | 0.994 | 0.321 | 0.009 |
|  |  |  |  |  |  |
| **BIS** | Target presence | 1, 111 | 3.801 | 0.054 | 0.033 |
|  | Target presence ✻ Prevalence | 1, 111 | 41.184 | < .001 | 0.271 |
|  | Absent Low - Absent High | 111 | 3.235 | 0.002 |  |
|  | Absent Low - Present Low | 111 | 3.172 | 0.002 |  |
|  | Absent High - Present High | 111 | -5.892 | < .001 |  |
|  | Present Low - Present High | 111 | -6.889 | < .001 |  |
|  | Target presence ✻ Target type | 1, 111 | 0.402 | 0.527 | 0.004 |
|  | Target presence ✻ Prevalence ✻ Target type | 1, 111 | 0.472 | 0.493 | 0.004 |
|  | Prevalence | 1, 111 | 8.390 | 0.005 | 0.070 |
|  | Target type | 1, 111 | 19.391 | < .001 | 0.149 |
|  | Prevalence ✻ Target type | 1, 111 | 0.930 | 0.337 | 0.008 |
|  |  |  |  |  |  |

*RTs*

We next examined RTs, again to check for prevalence effects as well as our predictions regarding snakes. Figure 3 presents the descriptive statistics for these comparisons; statistical results are presented in Table 2. The ANOVA revealed a significant main effect of Target Presence with participants being slower on target absent compared to target present trials. We also found a significant interaction between Target Presence and Prevalence. Subsequent t-tests revealed that in the low (compared to the high) prevalence condition participants were faster in the target absent trials, but they were slower on target present trials. The main effect of Target Type was also significant; in contrast to our expectations, participants were slower to find snakes compared to rabbits. All other effects were nonsignificant. Thus, while we found evidence for the standard effects of target prevalence, contrary to our predictions, performance was worse for snakes compared to rabbits.


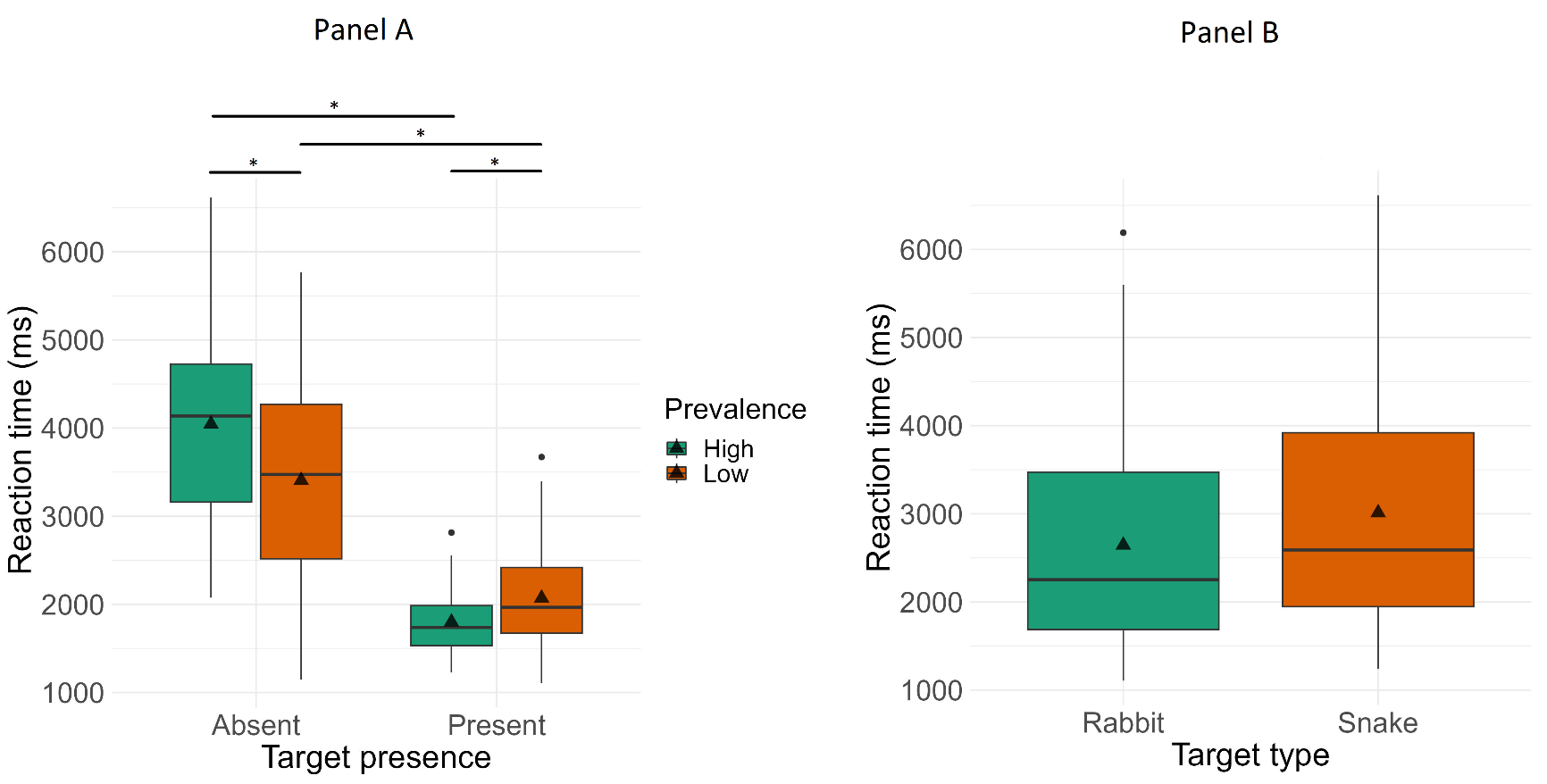


**Figure 3.** Reaction Times (in milliseconds) in Experiment 1 for low and high prevalence items (panel A) separately for target-present and target-absent trials and rabbit (neutral) and snake (threatening) targets (panel B) visualized as boxplots.

*BIS*

Finally, we examined the efficiency of performance (using BIS scores) to replicate standard prevalence effects and to test our prediction that prevalence effects would be less pronounced for snake compared to rabbit targets. Figure 4 presents the descriptive statistics for these comparisons; Table 2 shows all statistical results. We found that the main effect of target Prevalence was significant; finding targets in the low prevalence condition was less efficient compared to finding them in the high prevalence condition. The main effect of Target Type was also significant. Contrary to our prediction, finding snakes was less efficient compared to rabbits. The interaction between Prevalence and Target Presence was significant. In target-present trials, the high prevalence condition was more efficient compared to the low prevalence condition (as expected), while in target-absent trials the opposite was true. All other effects were nonsignificant.


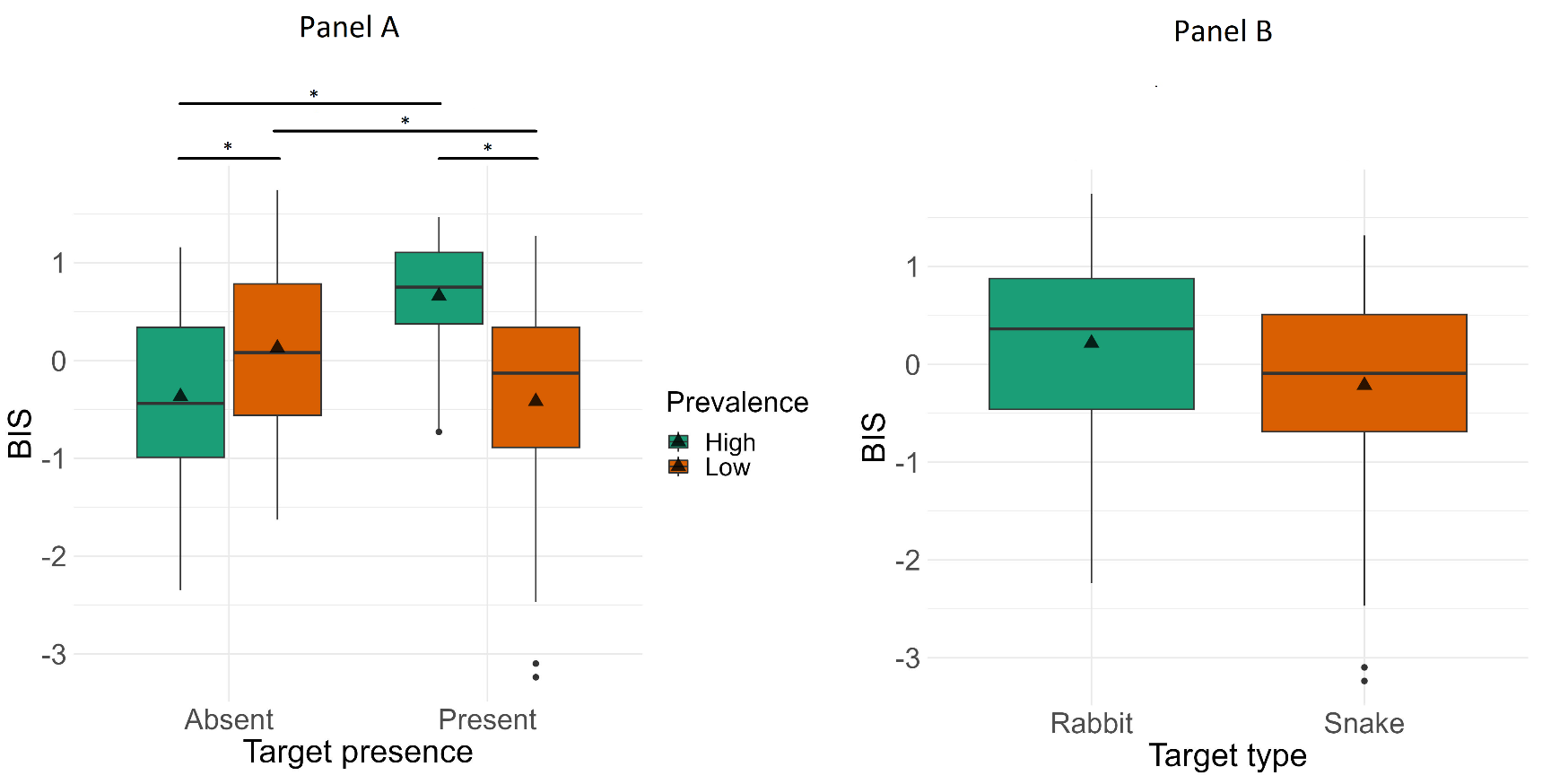


**Figure 4.** Balanced Integration Scores (BIS) in Experiment 1 for low and high prevalence items (panel A) separately for target-present and target-absent trials and rabbit (neutral) and snake (threatening) targets (panel B) visualized as boxplots.

Experiment 2

*Accuracy*

We began by examining response accuracy both to replicate standard effects of target prevalence and to test our prediction that the prevalence effect would be weaker for threatening compared to nonthreatening targets. Figure 5 presents the descriptive statistics for these comparisons; see Table 3 for the statistical results. Our ANOVA revealed a main effect of Target Presence, of Prevalence, and an interaction between Target Presence and Prevalence. Replicating the results of Experiment 1, in target-absent trials, accuracy was similar across conditions, while in target-present trials, participants were less accurate in the low compared to the high prevalence condition. However, here we also found a significant main effect of Target type. Participants identified snakes with significantly lower accuracy compared to rabbits while cockroaches and caterpillars did not significantly differ from either of these categories. All other effects were nonsignificant. Contrary to our predictions, response accuracy for threatening targets was not higher than for nonthreatening ones.


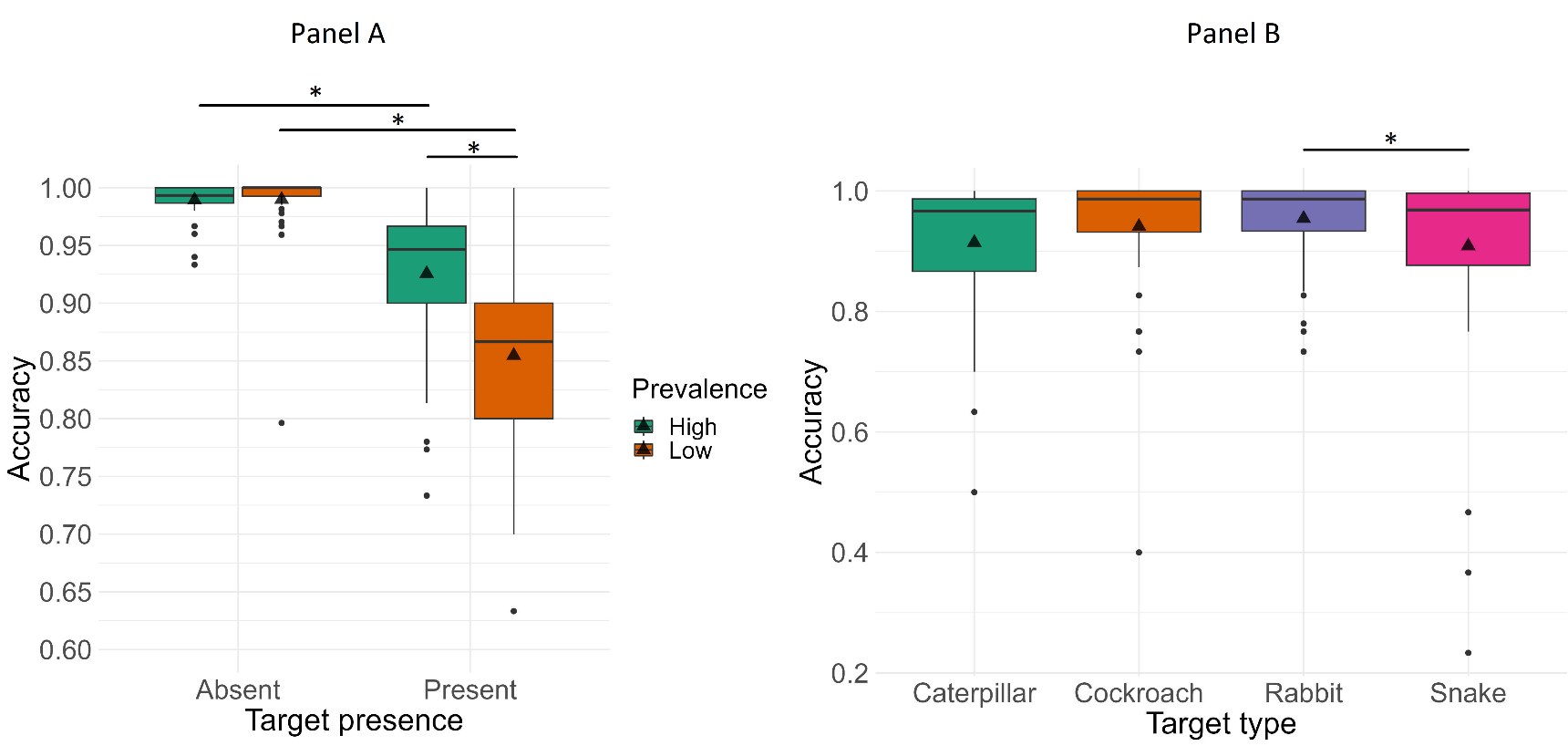


**Figure 5.** Accuracy in Experiment 2 for low and high prevalence items (panel A) and the four types of targets separately for target-present and target-absent trials (panel B) visualized as boxplots.

**Table 3**. Detailed statistical results for Experiment 2 (accuracy, reaction time, and BIS) with main effects, interactions, and follow-up simple effects.

| **Measure** | **Effect** | **df** | **F** | **p** | **η²p** |
| --- | --- | --- | --- | --- | --- |
| **Accuracy** | Target presence | 1, 101 | 115.41 | < .001 | 0.533 |
|  | Target presence ✻ Prevalence | 1, 101 | 24.99 | < .001 | 0.198 |
|  | Absent Low - Absent High | 101 | 0.267 | 0.790 |  |
|  | Absent Low - Present Low | 101 | 11.224 | < .001 |  |
|  | Absent High - Present High | 101 | 4.029 | < .001 |  |
|  | Present Low - Present High | 101 | -4.822 | < .001 |  |
|  | Target presence ✻ Target type | 3, 101 | 1.95 | 0.126 | 0.055 |
|  | Target presence ✻ Prevalence ✻ Target type | 3, 101 | 1.11 | 0.348 | 0.032 |
|  | Prevalence | 1, 101 | 20.34 | < .001 | 0.168 |
|  | Target type | 3, 101 | 3.34 | 0.022 | 0.090 |
|  | Rabbit - Snake | 101 | 2.879 | 0.025 | * |
|  | Rabbit - Caterpillar | 101 | 2.051 | 0.177 | * |
|  | Rabbit - Cockroach | 101 | 0.736 | 0.882 | * |
|  | Snake - Caterpillar | 101 | -0.732 | 0.884 | * |
|  | Snake - Cockroach | 101 | -2.098 | 0.161 | * |
|  | Caterpillar - Cockroach | 101 | -1.308 | 0.560 | * |
|  | Prevalence ✻ Target type | 3, 101 | 1.37 | 0.255 | 0.039 |
|  |  |  |  |  |  |
| **RT** | Target presence | 1, 101 | 382.101 | < .001 | 0.791 |
|  | Target presence ✻ Prevalence | 1, 101 | 43.349 | < .001 | 0.300 |
|  | Absent Low - Absent High | 101 | -4.00 | < .001 |  |
|  | Absent Low - Present Low | 101 | 9.24 | < .001 |  |
|  | Absent High - Present High | 101 | 18.33 | < .001 |  |
|  | Present Low - Present High | 101 | 2.34 | 0.021 |  |
|  | Target presence ✻ Target type | 3, 101 | 2.012 | 0.117 | 0.056 |
|  | Target presence ✻ Prevalence ✻ Target type | 3, 101 | 0.873 | 0.458 | 0.025 |
|  | Prevalence | 1, 101 | 4.86 | 0.030 | 0.046 |
|  | Target type | 3, 101 | 3.42 | 0.020 | 0.092 |
|  | Rabbit - Snake | 101 | -0.0823 | 1.000 | * |
|  | Rabbit - Caterpillar | 101 | 1.0590 | 0.715 | * |
|  | Rabbit - Cockroach | 101 | 2.7043 | 0.040 | * |
|  | Snake - Caterpillar | 101 | 1.1659 | 0.650 | * |
|  | Snake - Cockroach | 101 | 2.8527 | 0.027 | * |
|  | Caterpillar - Cockroach | 101 | 1.6143 | 0.375 | * |
|  | Prevalence ✻ Target type | 3, 101 | 1.61 | 0.192 | 0.046 |
|  |  |  |  |  |  |
| **BIS** | Target presence | 1, 101 | 5.021 | 0.027 | 0.047 |
|  | Target presence ✻ Prevalence | 1, 101 | 42.140 | < .001 | 0.294 |
|  | Absent Low - Absent High | 101 | 3.955 | < .001 |  |
|  | Absent Low - Present Low | 101 | 3.031 | 0.003 |  |
|  | Absent High - Present High | 101 | -6.124 | < .001 |  |
|  | Present Low - Present High | 101 | -5.253 | < .001 |  |
|  | Target presence ✻ Target type | 3, 101 | 1.791 | 0.154 | 0.051 |
|  | Target presence ✻ Prevalence ✻ Target type | 3, 101 | 0.605 | 0.613 | 0.018 |
|  | Prevalence | 1, 101 | 2.02 | 0.159 | 0.020 |
|  | Target type | 3, 101 | 5.65 | 0.001 | 0.144 |
|  | Rabbit - Snake | 101 | 2.311 | 0.102 | * |
|  | Rabbit - Caterpillar | 101 | 0.655 | 0.914 | * |
|  | Rabbit - Cockroach | 101 | -1.734 | 0.312 | * |
|  | Snake - Caterpillar | 101 | -1.603 | 0.382 | * |
|  | Snake - Cockroach | 101 | -4.065 | < .001 | * |
|  | Caterpillar - Cockroach | 101 | -2.357 | 0.092 | * |
|  | Prevalence ✻ Target type | 3, 101 | 1.54 | 0.210 | 0.044 |
|  |  |  |  |  |  |
| *Note: * means Tukey corrected p values* | |  |  |  |  |

*RTs*

We then examined RTs, again to check for prevalence effects as well as our predictions regarding the threat targets. Figure 6 presents the descriptive statistics for these comparisons; statistical results are presented in Table 3. The ANOVA revealed a significant main effect of Target Presence with participants being slower on target absent compared to target present trials. We also found a significant interaction between Target Presence and Prevalence. Pairwise comparisons revealed that in the low (compared to the high) prevalence condition participants were faster in the target absent trials, but they were slower on target present trials. The main effect of Target Type was also significant; in contrast to our expectations, RTs did not differ for finding snakes and caterpillars and rabbits. Participants found cockroaches faster than snakes and rabbits. All other effects were nonsignificant. While we found evidence for the standard effects of target prevalence, contrary to our prediction, performance was worse for threatening compared to nonthreatening targets.


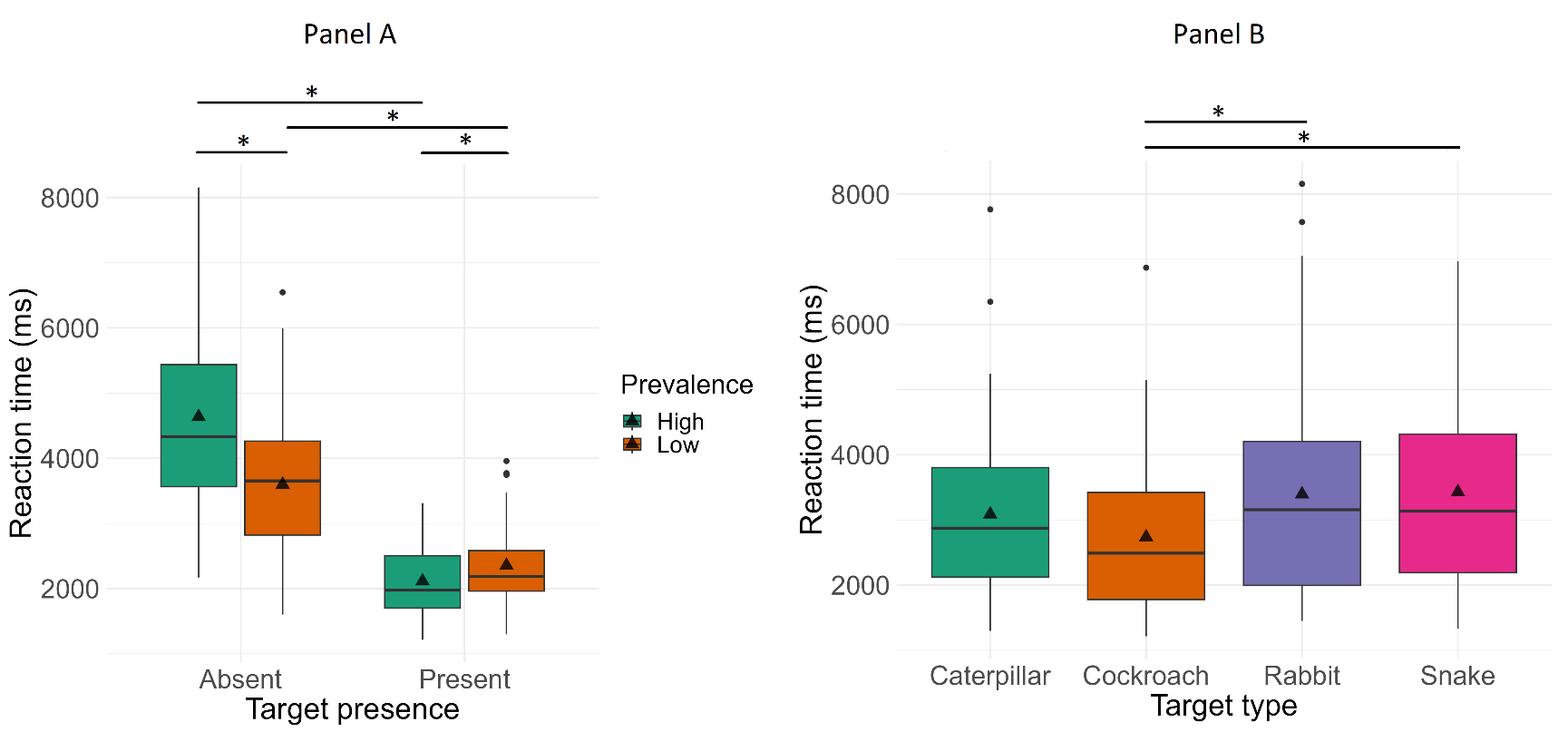


**Figure 6.** Reaction Times (in milliseconds) in Experiment 2 for low and high prevalence items (panel A) and the four types of target separately for target-present and target-absent trials (panel B) visualized as boxplots.

*BIS*

Finally, we examined efficiency of performance to replicate standard prevalence effects and to test our prediction that this effect will be less pronounced for snakes compared to other, nonthreatening targets (rabbits, cockroaches, and caterpillars). We found that the main effect of target Prevalence was significant; performance in the low prevalence condition was less efficient compared to the high prevalence condition. The interaction between Prevalence and Target Presence was significant. In target-present trials, the performance in the high prevalence condition was more efficient compared to the low prevalence condition (as expected), while in target-absent trials the opposite was true. The main effect of Target Type was also significant. Contrary to our prediction, we did not find differences in performance efficiency between snakes compared to rabbits and caterpillars (see Figure 7). Participants were more efficient finding cockroaches compared to snakes. All other effects were nonsignificant.


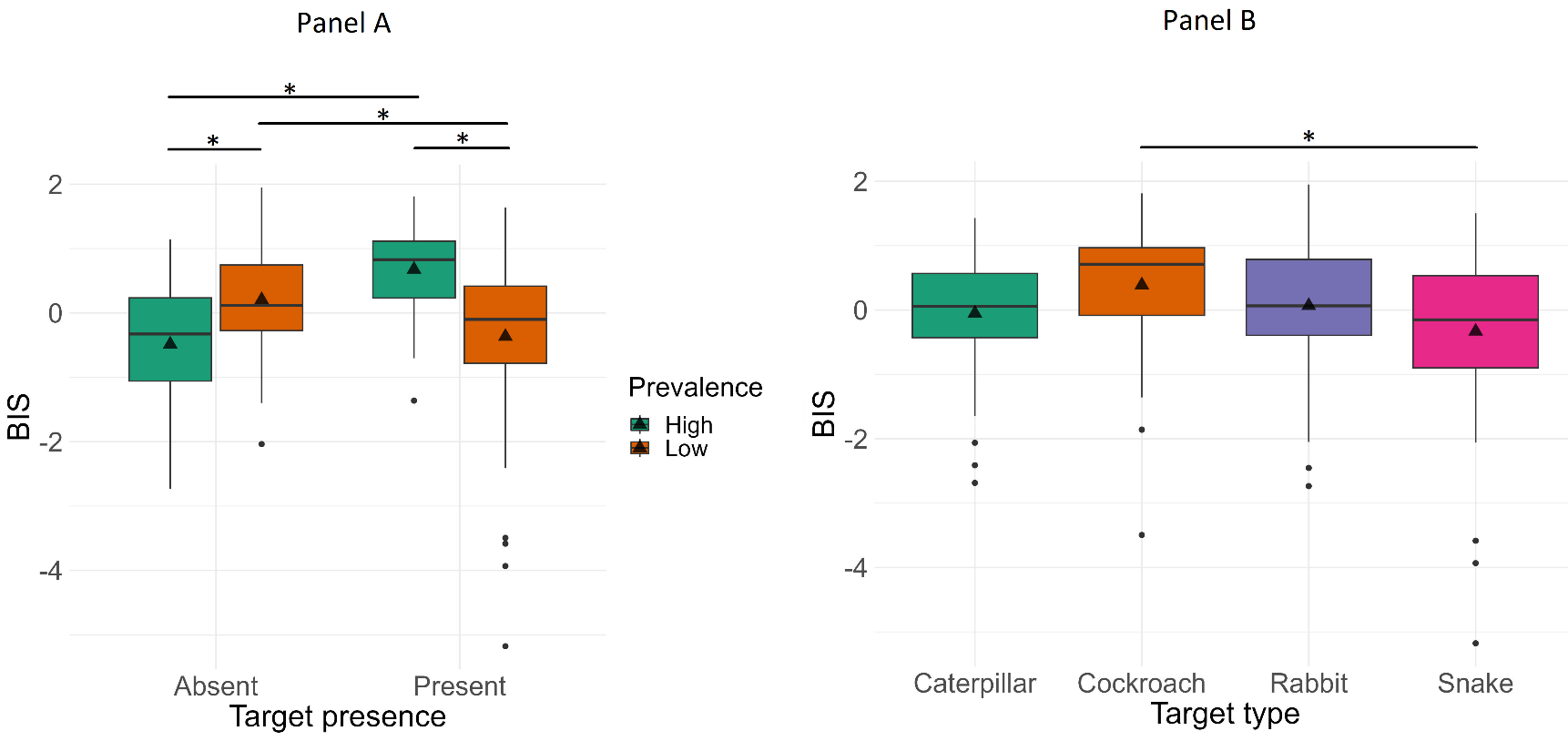


**Figure 7.** Balanced Integration Scores in Experiment 2 for low and high prevalence items (panel A) and the four types of targets separately for target-present and target-absent trials (panel B) visualized as boxplots.
