# Supplementary material for: No evidence of attentional prioritization for threatening targets in visual search
Source: Sci Rep. 2024 Mar 7;14:5651. doi: 10.1038/s41598-024-56265-1 (PMC10920919; doi:10.1038/s41598-024-56265-1)
Supplement: Supplementary file 2 — Supplementary Information 2. [file 41598_2024_56265_MOESM2_ESM.docx]

**Supplementary material 2**

Here we provide detailed descriptive statistics for all measurements across all manipulations. In both experiments, we measured accuracy (proportion correct), reaction time (in milliseconds), and balanced integration scores. These might not be vital parts of the manuscript, but the exact values might be useful for future research as a comparison.

**Supplementary Table 1.** Central tendencies for accuracy (proportion correct), reaction time (milliseconds), and balanced integration scores in Experiment 1 in each condition (Prevalence, Target Type, and Target Presence). Mean reaction times and 95% confidence interval (95% CI) values are presented in seconds.

|  |  |  |  |  | **95% CI** | |
| --- | --- | --- | --- | --- | --- | --- |
| **Measure** | **Prevalence** | **Target Type** | **Target Presence** | **Mean** | **Lower** | **Upper** |
| **Accuracy** | Low | Nonthreatening | Absent | 0.996 | 0.994 | 0.999 |
|  |  |  | Present | 0.837 | 0.800 | 0.874 |
|  |  | Threatening | Absent | 0.997 | 0.995 | 1.000 |
|  |  |  | Present | 0.804 | 0.769 | 0.840 |
|  | High | Nonthreatening | Absent | 0.994 | 0.991 | 0.996 |
|  |  |  | Present | 0.937 | 0.901 | 0.974 |
|  |  | Threatening | Absent | 0.995 | 0.993 | 0.998 |
|  |  |  | Present | 0.900 | 0.863 | 0.937 |
|  |  |  |  |  |  |  |
| **RTs** | Low | Nonthreatening | Absent | 3052 | 2633 | 3471 |
|  |  |  | Present | 1900 | 1732 | 2068 |
|  |  | Threatening | Absent | 3738 | 3333 | 4142 |
|  |  |  | Present | 2230 | 2068 | 2392 |
|  | High | Nonthreatening | Absent | 3923 | 3511 | 4334 |
|  |  |  | Present | 1692 | 1527 | 1857 |
|  |  | Threatening | Absent | 4173 | 3755 | 4592 |
|  |  |  | Present | 1911 | 1743 | 2079 |
|  |  |  |  |  |  |  |
| **BIS** | Low | Nonthreatening | Absent | 0.400 | 0.0850 | 0.7143 |
|  |  |  | Present | -0.154 | -0.4636 | 0.1562 |
|  |  | Threatening | Absent | -0.123 | -0.4265 | 0.1815 |
|  |  |  | Present | -0.663 | -0.9623 | -0.3635 |
|  | High | Nonthreatening | Absent | -0.284 | -0.5935 | 0.0249 |
|  |  |  | Present | 0.902 | 0.5970 | 1.2060 |
|  |  | Threatening | Absent | -0.453 | -0.7677 | -0.1383 |
|  |  |  | Present | 0.409 | 0.0995 | 0.7193 |
|  |  |  |  |  |  |  |

**Supplementary Table 2.** Central tendencies for accuracy (proportion correct), reaction time (milliseconds), and balanced integration scores in Experiment 2 in each condition (Prevalence, Target Type, and Target Presence). Mean reaction times and 95% confidence interval (95% CI) values are presented in seconds.

|  |  |  |  |  | **95% Confidence Interval** |  |
| --- | --- | --- | --- | --- | --- | --- |
| **Measure** | **Prevalence** | **Target Type** | **Target Presence** | **Mean** | **Lower** | **Upper** |
| **Accuracy** | Low | Dissimilar nonthreatening | Absent | 1.000 | 0.988 | 1.012 |
|  |  |  | Present | 0.882 | 0.817 | 0.947 |
|  |  | Threatening | Absent | 0.981 | 0.969 | 0.993 |
|  |  |  | Present | 0.738 | 0.675 | 0.801 |
|  |  | Similar nonthreatening | Absent | 0.984 | 0.972 | 0.995 |
|  |  |  | Present | 0.804 | 0.744 | 0.865 |
|  |  | Negative nonthreatening | Absent | 0.996 | 0.984 | 1.008 |
|  |  |  | Present | 0.833 | 0.768 | 0.899 |
|  | High | Dissimilar nonthreatening | Absent | 0.994 | 0.983 | 1.006 |
|  |  |  | Present | 0.939 | 0.876 | 1.002 |
|  |  | Threatening | Absent | 0.987 | 0.976 | 0.998 |
|  |  |  | Present | 0.916 | 0.857 | 0.975 |
|  |  | Similar nonthreatening | Absent | 0.982 | 0.969 | 0.996 |
|  |  |  | Present | 0.902 | 0.831 | 0.973 |
|  |  | Negative nonthreatening | Absent | 0.992 | 0.980 | 1.004 |
|  |  |  | Present | 0.943 | 0.877 | 1.008 |
|  |  |  |  |  |  |  |
| **RTs** | Low | Dissimilar nonthreatening | Absent | 3860 | 3145 | 4575 |
|  |  |  | Present | 2444 | 2127 | 2762 |
|  |  | Threatening | Absent | 3635 | 2946 | 4324 |
|  |  |  | Present | 2476 | 2170 | 2782 |
|  |  | Similar nonthreatening | Absent | 3458 | 2793 | 4124 |
|  |  |  | Present | 2230 | 1934 | 2526 |
|  |  | Negative nonthreatening | Absent | 3449 | 2734 | 4164 |
|  |  |  | Present | 2281 | 1963 | 2598 |
|  | High | Dissimilar nonthreatening | Absent | 5142 | 4453 | 5831 |
|  |  |  | Present | 2099 | 1793 | 2405 |
|  |  | Threatening | Absent | 5049 | 4404 | 5693 |
|  |  |  | Present | 2462 | 2176 | 2748 |
|  |  | Similar nonthreatening | Absent | 4634 | 3857 | 5412 |
|  |  |  | Present | 2198 | 1853 | 2544 |
|  |  | Negative nonthreatening | Absent | 3586 | 2871 | 4301 |
|  |  |  | Present | 1632 | 1314 | 1949 |
|  |  |  |  |  |  |  |
| **BIS** | Low | Dissimilar nonthreatening | Absent | 0.1276 | -0.357 | 0.6123 |
|  |  |  | Present | 0.1508 | -0.413 | 0.7144 |
|  |  | Threatening | Absent | 0.0874 | -0.380 | 0.5545 |
|  |  |  | Present | -1.0557 | -1.599 | -0.5126 |
|  |  | Similar nonthreatening | Absent | 0.2493 | -0.202 | 0.7005 |
|  |  |  | Present | -0.3463 | -0.871 | 0.1785 |
|  |  | Negative nonthreatening | Absent | 0.3560 | -0.129 | 0.8408 |
|  |  |  | Present | -0.1651 | -0.729 | 0.3986 |
|  | High | Dissimilar nonthreatening | Absent | -0.7877 | -1.255 | -0.3206 |
|  |  |  | Present | 0.7897 | 0.247 | 1.3329 |
|  |  | Threatening | Absent | -0.7704 | -1.207 | -0.3334 |
|  |  |  | Present | 0.3784 | -0.130 | 0.8865 |
|  |  | Similar nonthreatening | Absent | -0.5435 | -1.070 | -0.0166 |
|  |  |  | Present | 0.4368 | -0.176 | 1.0496 |
|  |  | Negative nonthreatening | Absent | 0.2374 | -0.247 | 0.7221 |
|  |  |  | Present | 1.1257 | 0.562 | 1.6893 |
|  |  |  |  |  |  |  |
